# Hierarchical Twin Networks Enable Exceptional Strength and Fracture Toughness in Titanium

**DOI:** 10.1002/advs.75805

**Published:** 2026-05-21

**Authors:** Xiao‐Wei Zou, Ting Zhu, En Ma, Wei‐Zhong Han

**Affiliations:** ^1^ Department of Materials Science and Engineering City University of Hong Kong Hong Kong P. R. China; ^2^ Center For Advancing Materials Performance from the Nanoscale State Key Laboratory For Mechanical Behavior of Materials Xi'an Jiaotong University Xi'an P. R. China; ^3^ George W Woodruff School of Mechanical Engineering Georgia Institute of Technology Atlanta Georgia USA; ^4^ Center for Alloy Innovation and Design State Key Laboratory for Mechanical Behavior of Materials Xi'an Jiaotong University Xi'an P. R. China; ^5^ Department of Materials Science and Engineering Department of Mechanical Engineering City University of Hong Kong Hong Kong P. R. China

**Keywords:** dislocation source, fracture resistance, hierarchical, titanium, twin boundary

## Abstract

Hexagonal close‐packed (HCP) metals such as titanium are widely used in aerospace applications, where simultaneously achieving high specific strength and fracture toughness is essential for operational safety. However, their intrinsically low symmetry crystal structures and the adverse effects of conventional toughening methods have long imposed a trade‐off between strength and fracture toughness. Here, we demonstrate that introducing pre‐engineered hierarchical twin networks endows commercial‐purity HCP titanium with an exceptional fracture toughness (*K_JIc_*) of ∼187 MPa·m^1/2^ and a specific yield strength of 140 MPa·cm^3^·g^−1^, its overall performance is superior to most titanium alloys and even some benchmark high‐toughness metals at room temperature. The activation and multiplication of <c+a> pyramidal dislocations, typically difficult in titanium, are remarkably promoted by the pre‐engineered high‐density twin network. The resultant profuse <c+a> and <a> dislocation activities substantially enhance crack‐tip plastic flow and suppress damage nucleation. This strategy is further validated in commercial‐purity HCP zirconium, achieving nearly a twofold improvement in both fracture toughness and yield strength. Our findings establish twin‐network engineering as a powerful microstructural design strategy for developing high‐performance HCP metals for safety‐critical applications.

## Graphical abstract

A commercial‐purity titanium with hierarchical ultrafine twin structures delivers an exceptional combination of fracture toughness (K_JIc_) of 187 MPa·m^1/^ ^2^ and specific yield strength of 140 MPa·cm^3^·g^−^^1^, in which pre‐engineered high‐density twin boundaries stimulate massive <a> and <c+a> dislocations to blunt crack tip.

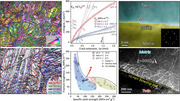

## 1 Introduction

Hexagonal close‐packed (HCP) metals such as titanium (Ti) are widely used in aerospace, marine, and biomedical applications, owing to their superior corrosion resistance, biocompatibility, and high specific yield strength (yield strength‐to‐density ratio) [1, 2]. As aerospace safety requirements continue to rise, next‐generation Ti components must not only achieve higher strength but, more crucially, exhibit superior damage tolerance, particularly in terms of fracture toughness [3, 4]. Conventional damage‐tolerant Ti alloys, such as α+β and metastable β types, typically improve fracture toughness through alloying. This involves introducing a high density of phase interfaces via the addition of expensive β‐stabilizers (e.g., Mo, V, Nb) to enhance extrinsic toughening mechanisms like crack deflection and bridging [4, 5, 6]. However, these mechanisms act only behind the crack tip and fail to address the intrinsic limitation of inadequate crack‐tip plasticity during crack initiation [6, 7, 8], a consequence of the low symmetry of HCP α‐Ti phase [9, 10, 11], thereby constraining overall toughening effectiveness [6, 12].

Overcoming the fracture toughness bottleneck primarily requires promoting intrinsic toughening mechanisms [6, 7, 8]−specially, achieving excellent uniform deformation ahead of the crack tip through the synergistic activation of <a>‐ and <c>‐axis deformation modes in HCP‐Ti, including <a> slip, <c+a> slip, and deformation twinning [13]. However, the intrinsic activation stresses for these deformation modes differ by several folds and are highly sensitive to interstitial elements such as oxygen [13, 14, 15]. Even trace amounts of oxygen atoms can markedly hinder <a>/<c+a> dislocation slip, promote strain localization, and suppress twinning under both uniaxial tension and crack‐tip triaxial stresses [16, 17], ultimately degrading ductility and fracture toughness [18, 19]. Therefore, a key toughening strategy involves reducing interstitial impurities, particularly oxygen, in Ti and its alloys, as demonstrated by the damage‐tolerant Ti‐6Al‐4 V Extra Low Interstitial (Ti‐6Al‐4 V ELI) alloy [20]. Unfortunately, this oxygen‐reduction toughening strategy inevitably compromises strength, since oxygen is the most potent solid‐solution strengthener in HCP‐Ti [13, 21, 22], resulting in the strength‐fracture toughness trade‐off [19, 20]. In addition, stringent control of oxygen impurities significantly increases processing costs of α and α+β Ti alloys, while alloying elements typically degrade crack‐tip plasticity, making it difficult to surpass the fracture toughness threshold of 130 MPa·m^1/2^ [19].

In this study, we demonstrate that the long‐standing trade‐off between strength and fracture toughness in HCP metals such as Ti can be effectively overcome by engineering multi‐variant twin architectures with ultrafine (submicron) twin thickness in commonly available commercial‐purity Ti (CP‐Ti), without relying on strict oxygen control or the addition of costly β‐stabilizing elements. Using cryogenic alternating directional rolling at 77 K—an easily scalable processing route—we introduce a high density of multiple (four‐variant) ultrafine deformation twins in CP‐Ti with 0.16 wt.% oxygen. Benefiting from the synergistic strengthening effect of oxygen and twin boundaries, the resulting Ti achieves a yield strength of 630 MPa (specific yield strength of 140 MPa·cm^3^·g^−1^), comparable to that of medium‐strength Ti alloys. More importantly, the pre‐existing high‐density twin networks near the crack tip act as prolific dislocation sources, activating extensive <a> and <c+a> dislocation activity. This mechanism mitigates oxygen‐induced embrittlement and suppresses crack initiation. As a result, the hierarchical ultrafine‐twinned Ti achieves an exceptional fracture toughness of 187 MPa·m^1/2^, which reaches an exceptional specific yield strength‐fracture toughness combination, surpassing most Ti alloys and even some benchmark high‐toughness metals at room temperature (RT). Additionally, our experiments confirm that introducing a hierarchical ultrafine‐twinned structure within grains also enhances both strength and fracture toughness in HCP‐zirconium (Zr). This twin‐network engineering strategy provides a new pathway for designing strong and damage‐tolerant HCP metallic materials.

## 2 Results and Discussion

### 2.1 Hierarchical Ultrafine‐Twinned Microstructure

**FIGURE 1:**
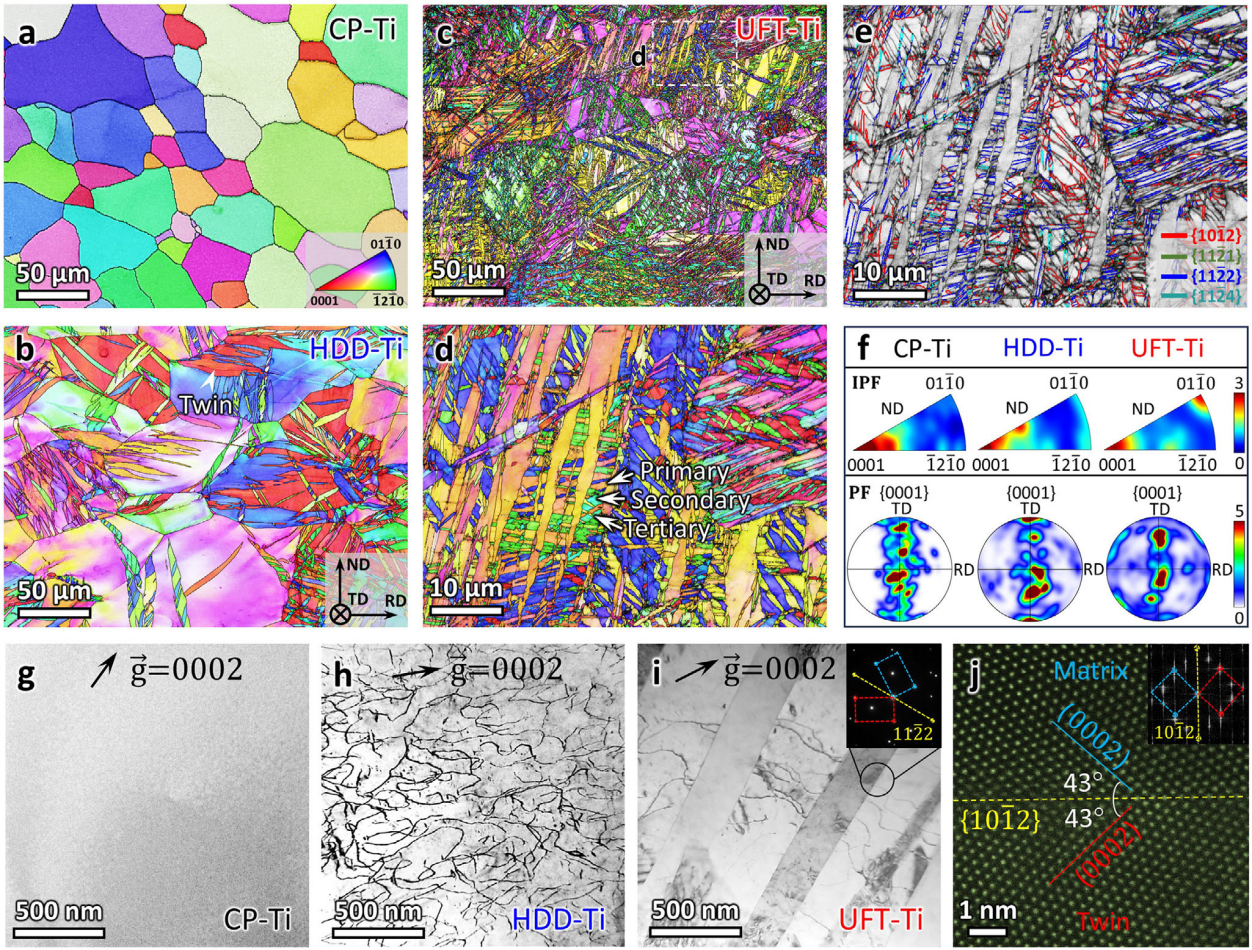
Microstructure of commercial‐purity Ti (CP‐Ti), high‐density dislocation Ti (HDD‐Ti), and hierarchical ultrafine‐twinned Ti (UFT‐Ti). a) Electron backscatter diffraction (EBSD) map showing the initial structure in CP‐Ti. b) HDD‐Ti exhibits the formation of some deformation twins. c–e) UFT‐Ti displays a hierarchical twinned architecture. A high density of deformation twins with multiple variants is observed in (e), while ultrafine secondary and tertiary twins are visible within the primary twins in (d). f) Inverse pole figure (IPF) and pole figure (PF) maps of CP‐Ti, HDD‐Ti, and UFT‐Ti indicate similar overall micro‐textures. g–i) TEM images showing that CP‐Ti contains no <c+a> dislocations, HDD‐Ti possesses a large number of <c+a> dislocations, and UFT‐Ti features ultra‐dense deformation twins together with some <c+a> dislocations. The inset in (i) shows the selected area diffraction pattern (SADP) of the {1$1\bar{2}$2} twin boundary. j) Aberration‐corrected high‐angle annular dark‐field scanning transmission electron microscopy (HAADF‐STEM) image of the {10$\bar{1}$2} coherent twin boundary.

CP‐Ti containing 0.16 wt.% oxygen (Table S1 for composition details) was selected as the base material, as twinning becomes prohibitively difficult even at 77 K when the oxygen content exceeds 0.2 wt.% [14]. The as‐received CP‐Ti has equiaxed grains with an average grain size of ∼30 µm (Figure 1a; Figure S3m) and contains no detectable deformation twins or dislocations of either <a> or <c+a> type (Figure 1g; Figure S15). Structural modification was achieved via either room‐temperature (RT) rolling, which primarily generates dislocations, or liquid‐nitrogen‐temperature (LNT) rolling, which promotes twin formation. The two sets of samples produced under these distinct conditions are hereafter referred to as high‐density dislocation Ti (HDD‐Ti) and hierarchical ultrafine‐twinned Ti (UFT‐Ti), respectively. Alternating directional deformation (Figure S1) mitigated the anisotropy inherent to the HCP structure by activating multiple deformation modes across different orientations, while the thickness reduction was limited to 20% per rolling direction to preserve the original grain morphology and orientation. Post‐rolling annealing at 450°C for 1 h effectively relieved residual stress without inducing recrystallization or grain coarsening (Figure S1).

After RT rolling, a limited number of deformation twins—primarily thick {10$\bar{1}$2} (T1) and {11$\bar{2}$2} (C1) variants—were observed within slightly elongated grains in HDD‐Ti (Figure 1b; Figure S11b). The average grain and twin sizes are ∼5 µm (Figure S3). Transmission electron microscopy (TEM) observations under the two‐beam condition of **g** = 0002 (Figure 1h; Figure S16) reveals substantial <c+a> dislocations alongside these twins, producing pronounced intragranular misorientations (Figure 1b). In contrast, UFT‐Ti exhibits nearly equiaxed grains containing hierarchical twins across multilength scales: micron‐scale primary twins (predominantly {10$\bar{1}$2} and {11$\bar{2}$2} types) containing profuse secondary twins with submicron‐scale thickness and tertiary twins with thickness down to ∼100 nm (Figure 1d). Grain boundary misorientation analysis (Figure 1e; Figure S12c) confirms the presence of additional {11$\bar{2}1$} (T2) and {11$\bar{2}$4} twin variants. TEM imaging identifies nanoscale {11$\bar{2}$2} twins (Figure 1i), while aberration‐corrected high‐angle annular dark‐field scanning transmission electron microscopy (HAADF‐STEM) resolves the atomic structure of a coherent {10$\bar{1}$2} twin boundary (Figure 1j). These hierarchical twins, occupying 78% of the areal fraction, form an ultra‐dense network with a twin boundary density of 2.23 µm/µm^2^ (total length of twin boundary per unit area) (Figures S3 and S13), in which <c+a> dislocations are observed in both the matrix (Figure 1i) and the twins (Figure S17a) under **g** = 0002. Electron backscatter diffraction (EBSD) analyses indicate comparable textures across CP‐Ti, HDD‐Ti, and UFT‐Ti, all showing preferential <c>‐axis alignment toward the sample normal direction (ND) and transverse direction (TD).

### 2.2 Extraordinary Mechanical Properties

**FIGURE 2:**
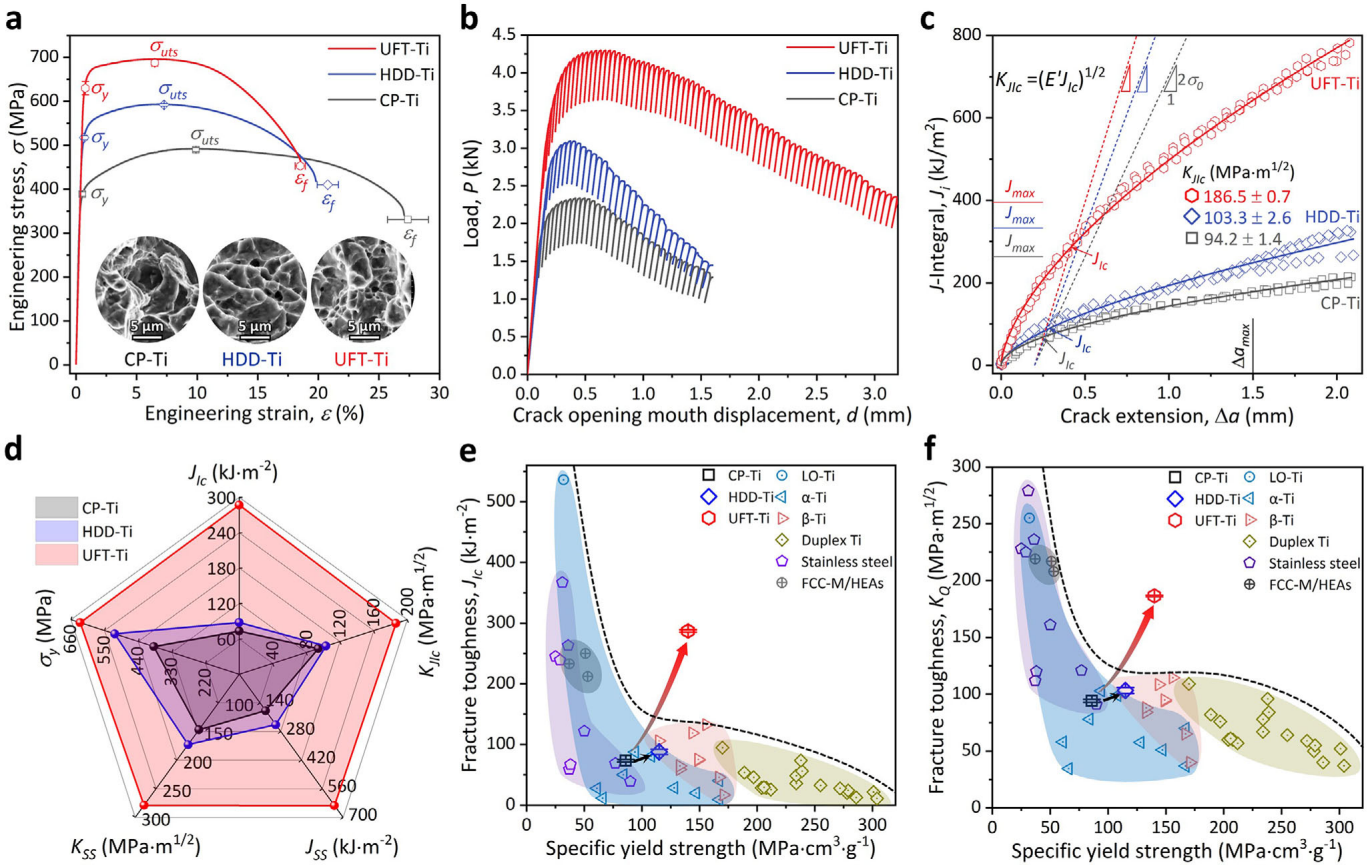
Tensile behavior and fracture toughness of CP‐Ti, HDD‐Ti, and UFT‐Ti. a) Engineering tensile stress‐strain curves, with insets showing SEM images of the corresponding fractured surfaces. b) Load‐crack mouth opening displacement curves of these three types of Ti, obtained from single‐edge notched bend (SENB) specimen tests. c) *J*‐integral‐based crack resistance (*J*–R) curves, with fracture toughness *K*_JIc_ values of 94.2, 103.3, and 186.5 MPa·m^1/2^ for CP‐Ti, HDD‐Ti, and UFT‐Ti, respectively. d) Comparison of yield strength and fracture toughness among the three Ti materials. e) Crack‐initiation toughness *J*_Ic_ and f) fracture toughness *K*_Q_ vs specific yield strength plots, showing that the overall performance of UFT‐Ti surpasses that of most pure Ti, Ti alloys [19, 25, 26, 27, 28, 29, 30, 31, 32, 33, 34, 35], and even some benchmark high‐toughness metals such as austenitic stainless steels and medium/high entropy alloys (M/HEAs) with face‐centered‐cubic structures [8, 36, 37, 38, 39, 40, 41].

Uniaxial tensile tests and three‐point bending mode I fracture toughness tests were performed at RT to evaluate the mechanical properties of the Ti materials. Prior to rolling, CP‐Ti exhibited a yield strength of 389 MPa and a total elongation of 27.4% (Table S3), consistent with the strengthening effects of oxygen solutes, which enhance strength but reduce ductility relative to low‐oxygen Ti [19]. The yield strength of HDD‐Ti increased to 517 MPa, attributable to the high density of RT‐rolling‐induced dislocations, whereas its elongation to failure decreased to 20.8%. The UFT‐Ti achieved a yield strength of 630 MPa with a corresponding specific yield strength of 140 MPa·cm^3^·g^−1^ (representing a 62% increase over CP‐Ti) through the formation of hierarchical ultrafine twins, yet maintained an elongation‐to‐failure of 18.5%, comparable to that of HDD‐Ti. All three materials exhibited fully ductile features, characterized by dimpled fracture surfaces after tensile failure (Figure 2a, inset). The average dimple diameters for CP‐Ti, HDD‐Ti, and UFT‐Ti are ∼2.7, 1.6, and 1.3 µm, respectively (Figure S6). The similar dimple diameter and depth of HDD‐Ti and UFT‐Ti suggest comparable capacities for uniaxial tensile deformation. Loading‐unloading‐reloading tensile tests were performed to measure the hetero‐deformation‐induced (HDI) back stress (Figure S4) [23]. The hierarchical ultrafine twin structure generated a relatively high back stress of ∼350 MPa (Figure S4d) and increased dislocation pile‐up (Figure S4e–g), both contributing to the enhanced strength.

Fracture toughness was evaluated using nonlinear elastic fracture mechanics by constructing *J*‐integral‐based crack‐resistance *R*‐curves (*J* vs. crack extension, Δ*a*), following the ASTM E1820 standard [24]. Tests employed single‐edge notched bend (SENB) specimens (6 mm thick × 12 mm wide) with side grooves (Figure S2). Load‐crack mouth opening displacement curves (Figure 2b) revealed rapid crack propagation in CP‐Ti and HDD‐Ti, evidenced by steep load drops [24], whereas UFT‐Ti exhibited a clear delay in both crack initiation and extension. The superior crack resistance of UFT‐Ti was further confirmed by its *J*–R curves (Figure 2c), which showed sharp *J*‐integral increases with crack growth, in stark contrast with CP‐Ti and HDD‐Ti. Crack‐initiation toughness *J*_Ic_ was determined from the intersection of the *R* curve with the 0.2 mm offset line (Figure 2c; Text S1). CP‐Ti exhibited the lowest plane‐strain crack‐initiation toughness (valid *J*_Ic_ = 73.3 kJ·m^−2^). HDD‐Ti showed a modest improvement (87.9 kJ·m^−2^), despite its high density of initial <c+a> dislocations. Remarkably, UFT‐Ti achieved a *J*_Ic_ of 287.0 kJ·m^−2^—approximately four times that of CP‐Ti and three times that of HDD‐Ti. By *J*‐*K* equivalence, the stress‐intensity fracture toughness (*K_JIc_*) of UFT‐Ti reached 186.5 MPa·m^1/2^ (Figure 2c), nearly double that of CP‐Ti (94.2 MPa·m^1/2^) and HDD‐Ti (103.3 MPa·m^1/2^). The SENB specimen thickness exceeds the critical dimension (see details in Experimental Section; Table S3), and the negligible cross‐sectional shrinkage observed in the fractured SENB specimens (Figure S7) confirms the validity of plane‐strain condition for all samples, ensuring full compliance with the ASTM E1820 testing standard [24]. As shown in Figure 2d, UFT‐Ti achieves the best strength‐toughness matching compared to CP‐Ti and HDD‐Ti. In addition, a direct comparison of specific yield strength‐fracture toughness at RT (Figure 2e,f) demonstrates that the overall mechanical performance of UFT‐Ti outperforms that of most reported pure Ti and Ti alloys [25, 26, 27, 28, 29, 30, 31, 32, 33, 34, 35], as well as some benchmark high‐toughness metals, including austenitic stainless steels and face‐centered cubic (FCC) structured medium‐ or high‐entropy alloys (M/HEAs) [8, 36, 37, 38, 39, 40, 41]. Obviously, the hierarchical twin architecture, extending down to ultrafine scales, successfully pushes the boundary of the strength‐toughness trade‐off in metals [6].

### 2.3 Extensive Crack‐Tip Plasticity

**FIGURE 3:**
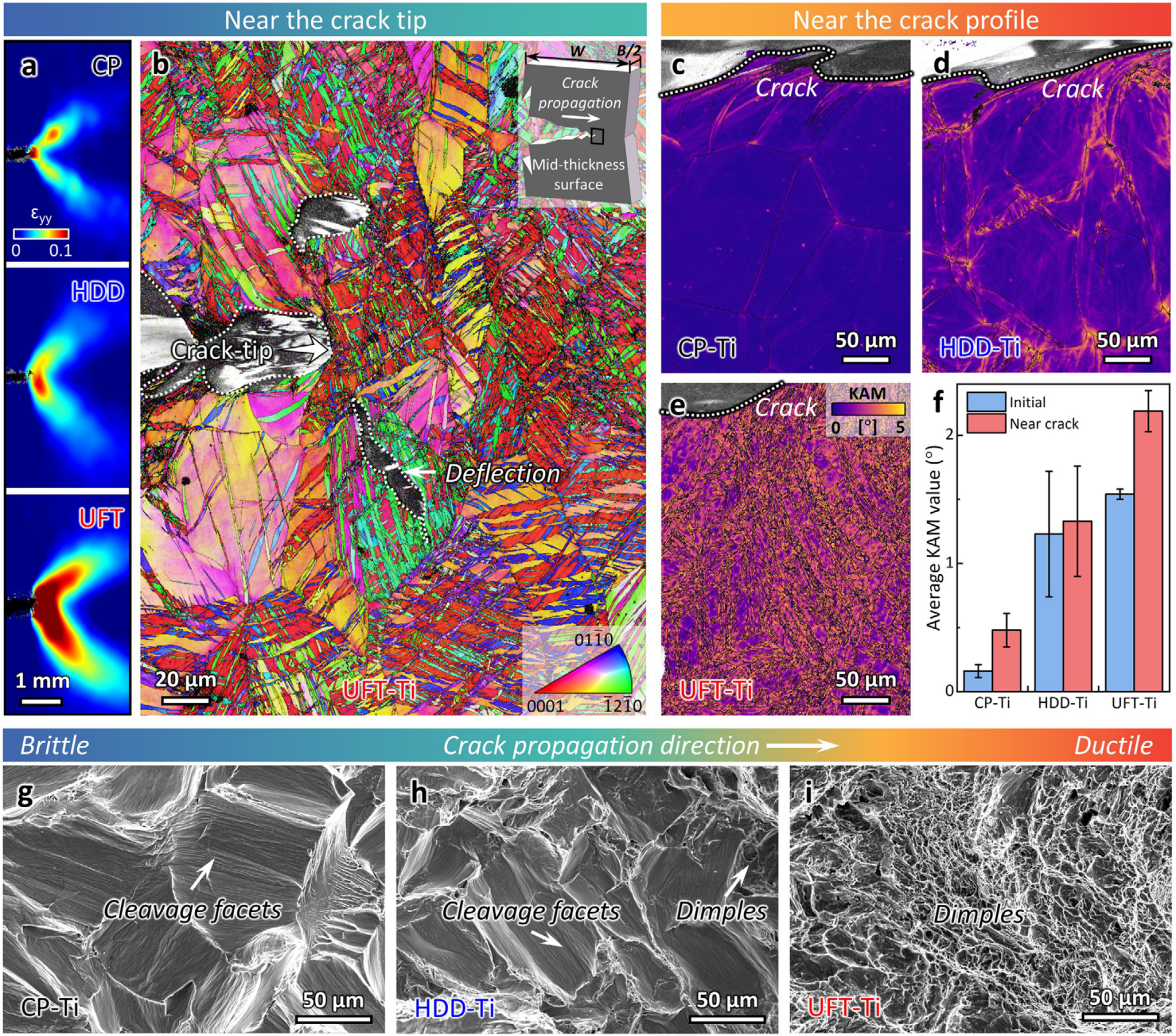
Cracking behaviors of CP‐Ti, HDD‐Ti, and UFT‐Ti. a) 2D strain distribution near the crack tip on the sample surface at crack initiation (crack extension ∆*a* ≈ 0.2 mm). b) EBSD IPF map of UFT‐Ti near the crack tip at the mid‐thickness plane (∆*a* ≈ 2 mm). c–e) EBSD kernel average misorientation (KAM) maps of CP‐Ti, HDD‐Ti and UFT‐Ti beneath the crack profile at ∆*a* ≈ 2 mm. f) Comparison of average KAM values before and after cracking showing that UFT‐Ti undergoes the greatest degree of plastic deformation ahead of the crack tip during crack propagation. g–i) SEM images of fracture surfaces showing semi‐brittle fracture modes with a mixture of cleavage facets and dimples in (g) CP‐Ti and (h) HDD‐Ti, whereas (i) UFT‐Ti exhibits a fully ductile fracture with densely distributed fine dimples. The stress intensity factor (*K_i_*) at the crack tip for CP‐Ti, HDD‐Ti, and UFT‐Ti is ∼ 41, 56, 68 MPa·m^1/2^, respectively, at (a) crack initiation (∆*a* ≈ 0.2 mm), and ∼ 40, 51, 67 MPa·m^1/2^, respectively, at (b–e) a crack extension of ∆*a* ≈ 2 mm.

To elucidate the synergistic strengthening and toughening mechanisms, we systematically investigated the deformation and fracture responses of CP‐Ti, HDD‐Ti, and UFT‐Ti SENB specimens. The evolution of surface plastic zones and the extent of plastic deformation at crack tips were quantified via in situ digital image correlation (DIC) [42], revealing pronounced contrasts in strain distribution at crack initiation (near peak load). In CP‐Ti and HDD‐Ti, the strain was confined within ∼200 µm of the crack tip, whereas UFT‐Ti developed an extended plastic zone exceeding 700 µm, accompanied by higher plastic strains (Figure 3a). SEM analysis of pre‐polished specimens at a crack extension of Δ*a* ≈ 2 mm (Figure S8) further corroborates this difference—UFT‐Ti exhibited homogeneously distributed and intense plasticity, in contrast to the localized and limited plasticity in CP‐Ti and HDD‐Ti. Interestingly, twin‐matrix domains in UFT‐Ti showed high‐density slip traces (Figure S8k,l), indicating that hierarchical twin networks can enhance crack‐tip plasticity by facilitating dislocation glide within both matrix and fine‐twin regions.

Post‐fracture side‐grooved specimens were bisected through the thickness to probe plane‐strain deformation mechanisms. CP‐Ti displayed sparsely activated deformation twins near the crack tip and along the propagation path (Figures S9–S11), consistent with oxygen‐induced suppression of deformation twinning at RT [14, 15]. Correspondingly, the crack‐tip opening displacement (*CTOD*) remains minimal at Δ*a* ≈ 2 mm (Figure S9a). HDD‐Ti, characterized by pre‐existing dislocations and limited twins, also produced low *CTOD* (Figure S9d), confirming ineffective toughening. In contrast, UFT‐Ti showed a *CTOD* value nearly three times that of CP‐Ti and HDD‐Ti at the same Δ*a* (Figure S9g). According to the *J*‐*CTOD* equivalence relation (*J* ∝ 𝜎_0_ *CTOD*) [38], this elevated *CTOD* reflects the exceptional fracture resistance of UFT‐Ti. Mechanistically, this behavior arises from pre‐existing ultrafine‐twin networks, which facilitate distributed dislocation slip at the crack tip (Figure S8k), thereby mitigating localized rupture and enhancing overall energy dissipation.

EBSD IPF maps reveal that a substantial fraction of deformation twins are retained near the crack tip (Figure 3b) and along the crack path (Figure S11f) in UFT‐Ti. Frequent crack path deflections (Figure 3b; Figure S9h) reduce crack driving force, thereby enhancing crack‐propagation toughness. However, this mechanism alone cannot account for the superior crack‐initiation toughness, which is typically closely associated with the intrinsic plasticity at the crack tip [6, 43, 44]. In HCP metals, both deformation twinning and dislocation slip can contribute to crack‐tip plasticity. To evaluate the role of deformation twinning or detwinning in crack‐tip deformation, the twin boundary density near the crack in a cracked sample with a crack extension of 2 mm was compared to that in the initial state. Notably, in UFT‐Ti, the twin boundary density, twin types, and twin size remained largely unchanged before and after cracking (Figures S12 and S13), confirming that pre‐existing twins at the crack tip hardly induced additional twinning or detwinning during crack propagation under triaxial stress conditions at RT, which can be attributed to the significant suppressive effect of oxygen solutes on deformation twinning [14, 18]. KAM analysis was further employed to highlight the difference in plasticity: CP‐Ti (Figure 3c) and HDD‐Ti (Figure 3d) shows localized grain‐boundary strain, whereas UFT‐Ti exhibits widespread, high‐intensity plasticity (Figure 3e). UFT‐Ti also demonstrates the largest absolute and relative KAM increase after cracking (Figure 3f), confirming that hierarchical ultrafine‐twin networks facilitate massive dislocation activity under plane‐strain constraints. Therefore, this intrinsic strain‐hardening capacity—driven by coordinated dislocation‐twin interactions—directly enhances crack‐initiation toughness by ∼100% compared to CP‐Ti, establishing dislocation‐mediated plasticity as the dominant toughening mechanism [6].

The distinct plastic deformation responses under crack‐tip triaxial stress states are clearly manifested in fracture surface topographies. CP‐Ti and HDD‐Ti display semi‐brittle fracture surfaces dominated by cleavage facets with sparse dimples (Figure 3g,h; Figure S7), consistent with limited crack‐tip plasticity. In contrast, UFT‐Ti's ultrafine twin architecture enables a fully ductile fracture mode characterized by uniformly distributed, high‐density dimples (Figure 3i; Figure S7). This morphological transition originates from twin‐induced dislocation multiplication: as detailed in the next section, twin boundaries activate multiple slip systems that facilitate strain delocalization (Figure S8k,l), transforming localized cleavage into extensive plastic dissipation under crack‐tip triaxial stresses.

Notably, the twin‐network strategy is also effective in HCP‐Zr. Introducing a hierarchical ultrafine‐twinned structure significantly improved the intrinsically constrained crack‐tip plasticity (Figures S22 and S24, Text S1), leading to concurrent enhancements in both yield strength and fracture toughness (Figure S23 and Table S5), compared to commercial‐purity Zr (CP‐Zr) with a coarse‐equiaxed grain structure.

### 2.4 <c+a> Dislocation Activity Enhanced by Twin Boundaries

Given the negligible twinning‐induced plasticity (TWIP) effect observed in all three types of Ti samples (Figures S12 and S13) [9, 45], the distinct plastic deformation capacities under crack‐tip triaxial stresses primarily originate from differences in dislocation activity. TEM analysis comparing dislocation structures in pre‐cracked samples and those with crack extension of ∆*a* ≈ 1 mm (using **g·b** analysis, where **g** is the diffraction vector and **b** is the Burgers vector) revealed that no <a> (**b** = 1/3<11$\bar{2}$0>) or <c+a> (**b** = 1/3<11$\bar{2}$3>) dislocations existed in the initial CP‐Ti (Figure S15a–c). Crack propagation activated abundant <a> dislocations but only sparse <c+a> types, as evidenced by dislocation visibility transitions under **g** = 0$\bar{1}$11→**g** = 0002 (Figure S15d–f), consistent with the much higher critical resolved shear stress (CRSS) required for <c+a> dislocation slip [46, 47]. The as‐received HDD‐Ti contained high density of both <a> and <c+a> dislocations (Figure S16a–d), but many <c+a> dislocations were aligned along pyramidal‐basal plane intersections (Figure S16c,d) with dominant edge components, exhibiting low mobility [48, 49, 50, 51]. Post‐cracking, the <c+a> dislocation density in HDD‐Ti remained largely unchanged (Figure S16e–h), as corroborated by nearly constant geometrically necessary dislocation densities (Figure S14i). This result indicates that pre‐existing <c+a> dislocations possess limited self‐multiplication capability at the crack tip. The inefficiency of dislocation multiplication stems from the large mobility difference between edge and screw dislocation components (Figure S21, Text S1) [52, 53].

**FIGURE 4:**
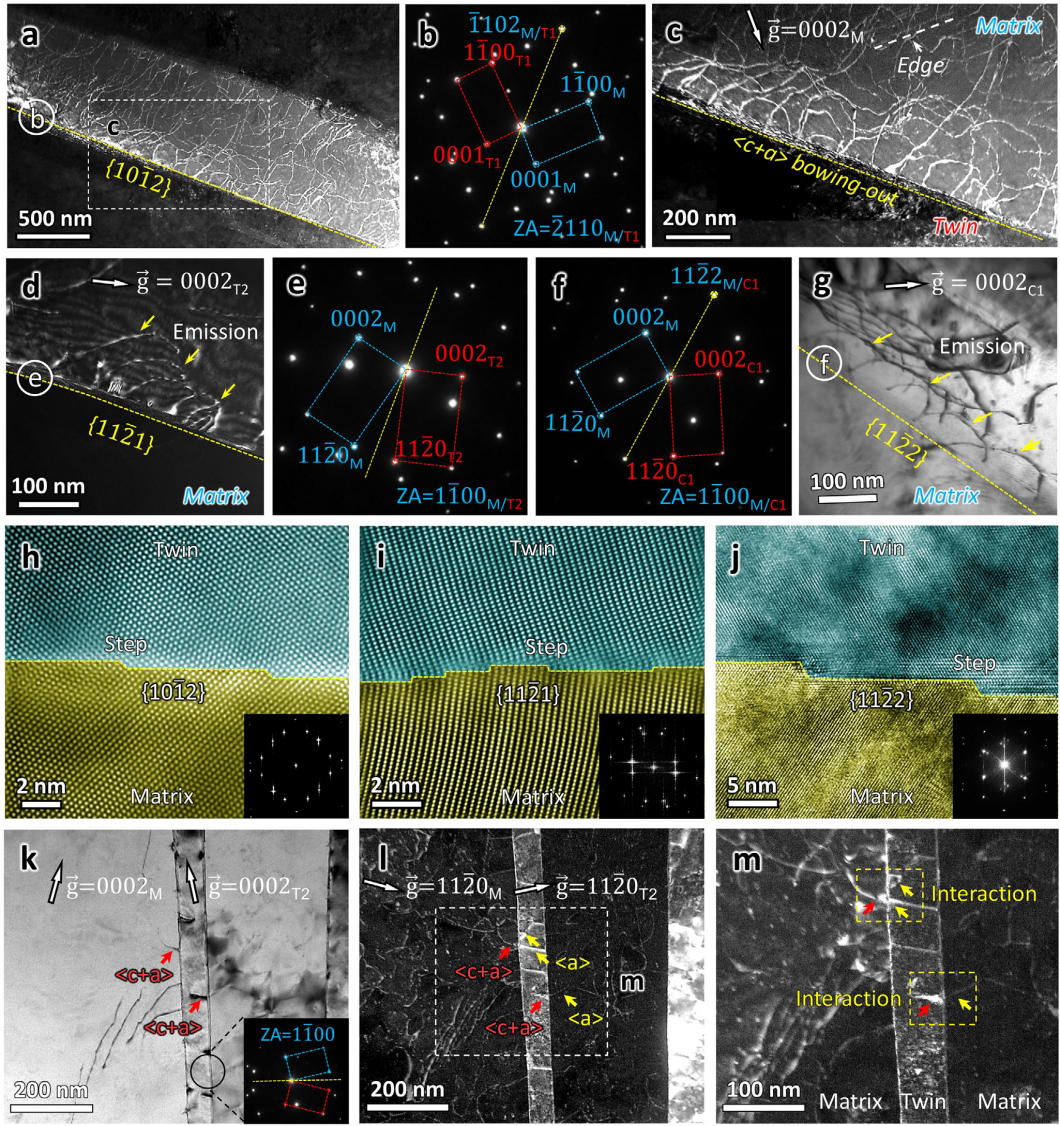
Deformation mechanisms in UFT‐Ti ahead of the crack tip (within the plastic zone) at a crack extension of ∆*a* ≈ 1 mm. a–g) TEM images showing that numerous <c+a> dislocations are emitted from the {10$\bar{1}$2} (T1), {11$\bar{2}$1} (T2), and {11$\bar{2}$2} (C1) twin boundaries, bowing out into both the matrix and twin regions. h–j) HAADF‐STEM images showing a high density of atomic‐scale steps along the {10$\bar{1}$2}, {11$\bar{2}$1}, and {11$\bar{2}$2} twin boundaries, which act as potential dislocation sources. Insets show the corresponding fast Fourier transform (FFT) patterns. k, l) Bright‐field and dark‐field TEM images showing that <c+a> dislocations are connected with <a> dislocations at a {11$\bar{2}$1} extension twin boundary. m) Enlarged dark‐field TEM image of the white square region in (*l*), highlighting the interconnection between <c+a> and <a> dislocations. The stress intensity factor (*K_i_*) at the crack tip for UFT‐Ti is ∼71 MPa·m^1/2^ at a crack extension of ∆*a* ≈ 1 mm.

Prior to crack propagation in UFT‐Ti, <a> dislocations predominated due to their lower activation stress [46, 47], although substantial <c+a> dislocation activity also occurred (Figure S17a–f). At crack extension Δ*a* ≈ 1 mm, numerous <c+a> edge dislocations were still observed (Figure S17h), exhibiting reduced mobility relative to screw counterparts due to dissociation along the basal plane (Figure S17e). This again demonstrates the limited self‐multiplication capability of pre‐existing <c+a> dislocations (Figure S21). Remarkably, a very different scenario emerged near the crack tip, where <c+a> dislocation activity was significantly intensified in both the matrix and twins (Figure 4a–g; Figure S17g–k), suggesting the presence of additional multiplication pathways. Detailed characterization revealed a high density of <c+a> dislocations near the boundaries of various twin variants ({10$\bar{1}$2}, {11$\bar{2}$1}, and {11$\bar{2}$2}). All these dislocations exhibit pronounced “bowing‐out” morphology on both the twin and the matrix sides (Figure 4a,c,d,g; Figure S17g–k), which is a direct sign of twin boundary as dislocation sources, as confirmed by in situ TEM study in nanotwinned Cu [54]. These observations establish twin‐boundary‐mediated dislocation nucleation as a key toughening mechanism in UFT‐Ti.

Fracture toughness fundamentally depends on intrinsic toughening mechanisms that promote uniform crack‐tip plastic deformation [6, 55]. However, the low‐symmetry crystal structure of HCP‐Ti inherently restricts the number of available slip systems [56]. While prismatic <a> slip with a low CRSS accommodates deformation along the <a>‐axis [46, 47], <c+a> dislocation slip is essential because it provides strain accommodation along the <a> and <c>‐axes—offering five independent systems required to satisfy the Taylor–von Mises criterion [19, 57, 58]. However, the scarcity of <c+a> dislocations at RT stems from their high activation stress [46, 47], as experimentally confirmed in CP‐Ti, compounded by the large mobility difference between edge and screw components, which further limits their self‐multiplication efficiency [52], as observed in HDD‐Ti. These two factors account for the limited <c+a> dislocation activity and consequently, the low fracture toughness in both CP‐Ti and HDD‐Ti.

The superior mechanical performance of UFT‐Ti lies in its twin network architecture, which actively emit <c+a> dislocations into both the matrix and twins, generating exceptional resistance to crack advancement. This is beyond traditional CRSS‐based activation mechanism. Previous studies have correlated increased c/a ratios in HCP‐Ti with reduced <c+a> slip CRSS [59, 60], attributed to the enhanced atomic packing density on pyramidal plane. Evidence supporting this relationship is observed in our twin regions, which exhibit elevated c/a ratios relative to the matrix, as determined by lattice spacing analysis (Figure S18). This change in c/a ratio can be attributed to the residual twinning dislocations produced during LNT rolling, whose *c*‐axis components contribute to the increase in axial ratio within twins [59]. Nevertheless, such a slight increase in c/a ratio could not exert a strong effect on <c+a> dislocation activities within the twins. In contrast, our observations reveal substantial <c+a> dislocation populations in both the matrix and twin regions under high crack‐tip stresses (Figure 4a–g; Figure S17g–k). This apparent contradiction suggests that beyond locally modifying slip thresholds through c/a ratio variations, twin boundaries play an additional, critical role in actively injecting <c+a> dislocations into the adjacent matrix and twin regions.

To elucidate the mechanisms of twin boundary‐driven <c+a> dislocation multiplication, atomic‐scale characterization of various twin boundaries near the crack tip was performed. The intrinsic elastic anisotropic of HCP lattices leads to distinct elastic and plastic responses between the matrix and the twin domains, which likely gives rise to strain incompatibility at the twin interfaces. This incompatibility is expected to induce stress concentration, which may assist the activation of <c+a> dislocations. In addition, these twin boundaries exhibit non‐planar morphologies with atomic‐level sharp steps along the {10$\bar{1}$2}, {11$\bar{2}$1}, and {11$\bar{2}$2} interfaces (Figure 4h–j). Such steps are recognized as effective nucleation sites for <c+a> dislocations [61, 62]. Notably, considering the triaxial stress conditions at the crack tip, combined with the uniform and random 3D distribution of ultrafine twins and twin boundaries, the orientation dependence of strain incompatibility across the twin boundary should not be a critical factor that affects the <c+a> dislocation behaviors. Previous studies have shown that <c>‐component twinning dislocations in Ti play a critical role in twin growth, leaving residual atomic steps in their wake [59]. In addition, TEM analyses have demonstrated that interactions between <a> dislocations and twin boundaries in HCP metals can generate both <c+a> dislocations within the twins and twinning dislocations [63], a mechanism clearly active in UFT‐Ti and occurs simultaneously in both sides of the twin boundaries (Figure 4; Figure S19). Specifically, multiple <a> dislocations within both the twins and the matrix interacting with twin boundaries were observed to undergo sequential decomposition and reorganization, leading to the nucleation of perfect <c+a> and twinning dislocations at the crack tip (Figure S19d–g). Spatial correlations between <a> dislocations (yellow arrows) and <c+a> dislocations (red arrows) across twin boundaries provide direct evidence of dislocation‐twin boundary reactions within both the twins and the matrix (Figure 4k–m; Figure S19a–c, Text S1). The subsequent accumulation of twinning dislocations promotes step formation at the boundaries (Figure 4h–j) and results in bent twin interfaces (Figures S10c and S11f) [54, 63]. Collectively, the abundance of twin‐boundary steps and the frequent <a> dislocation‐twin boundary interactions facilitate prolific nucleation of <c+a> dislocations, which subsequently glide into both the matrix and twin regions. These coupled mechanisms account for the extensive <c+a> dislocation activity observed in the plastic zone ahead of the crack tip in UFT‐Ti.

### 2.5 Twin Boundary–Stimulated <c+a> Dislocations for Strengthening and Toughening

High strength and high fracture toughness are crucial requirements for Ti and its alloy in engineering application. While unalloyed Ti exhibits exceptional fracture toughness, its low strength (<150 MPa) limits its structural applications [19]. Small amounts of oxygen solutes could significantly enhance the yield strength of Ti. For example, CP‐Ti with 0.16 wt.% oxygen in this study achieved a yield strength of 389 MPa (Table S3). However, oxygen solutes significantly suppress *c*‐axis deformation mode (e.g., twinning and <c+a> dislocation slip) at the crack tip (Figures S12 and S15), resulting in limited uniform plasticity and a low fracture toughness of 94 MPa·m^1/2^ (Table S3) [6, 19]. RT rolling increases dislocation density, which can elevate the yield strength to 517 MPa. However, the lack of an obvious increase in twin density or <c+a> dislocations after cracking (Figures S12 and S16) indicates limited <c>‐axis plasticity, leading to a fracture toughness of 103 MPa·m^1/2^ (Table S3). Cryorolling, on the other hand, creates an ultrafine twin structure, further raising the yield strength to 630 MPa and mitigating the negative effects of oxygen solutes on fracture toughness at RT. Pre‐existing twin boundaries efficiently promote <c+a> dislocations nucleation and multiplication (Figure 3; Figure S17), thereby facilitating cooperative plastic deformation between the *a*‐axis and *c*‐axis [62], improving crack‐tip uniform deformation and significantly enhancing fracture toughness.

Previous studies have demonstrated that the introduction of twin structures in Ti contributes to a synergistic enhancement of both strength and tensile ductility [9, 59]. The increase in c/a ratio owing to residual twinning dislocations, together with the interaction of <a> dislocations in matrix with twin boundaries, serve as two main drivers for activating <c+a> dislocations in the twin regions [59, 63]. However, under the crack‐tip triaxial stress conditions, pre‐existing twin boundaries have additional influence on the multiplication of <c+a> dislocations. Numerous <c+a> dislocations are activated in both the matrix and the twin regions (Figure 4a–g; Figure S17). Our study shows that <a> dislocations in both matrix and twin can interact with various types of twin boundaries to form twin‐boundary steps (Figure 4h–m; Figure S19), leading to the generation of numerous <c+a> dislocations from the twin boundaries, which are then emitted into both the matrix and the twin regions (Figure 4a–g). Numerous <c+a> dislocations induced by twin boundaries enable sufficient *a*‐axis and *c*‐axis plasticity, facilitating intense uniform deformation at the crack tip and ultimately increasing the fracture toughness of UFT‐Ti to 187 MPa·m^1/2^ (Table S3).

The twin boundary‐stimulated <c+a> dislocations effectively overcome the long‐standing strength‐fracture toughness trade‐off in pure Ti and Ti alloys. The strategy of strengthening and toughening through pre‐engineered twin structures is also applicable to HCP Zr (Table S5) and has potential implications for the development of twinning‐dominated metastable β/β‐Ti/Zr alloys as well. By incorporating specific elements, these alloys can achieve low elastic moduli, outstanding corrosion resistance, and biocompatibility, while also displaying favorable strength‐ductility synergy because of the twinning‐dominated deformation mode [64, 65, 66, 67, 68].

## 3 Conclusion

Achieving a strength‐toughness synergy in HCP metals such as Ti requires a mechanism that not only strengthens the material but also enables <c>‐axis deformation, a critical yet traditionally elusive objective. In HDD‐Ti, although a high density of <c+a> dislocations is introduced, their inefficient multiplication limits crack‐tip <c>‐axis plasticity. In contrast, UFT‐Ti fulfills this requirement through the formation of hierarchical twin networks. Upon interacting with <a> dislocations, these high‐density twin boundaries generate atomically sharp steps that act as potent sources for <c+a> dislocation emission. This mechanism substantially amplifies <c+a> dislocation activity near the crack tip, promoting uniform deformation and effective strain delocalization. The resulting expanded plastic zone and extensive plastic flow enhance crack‐tip plastic energy dissipation, thereby elevating the fracture resistance of CP‐Ti to unprecedented levels. Compared with conventional Ti alloys, UFT‐Ti offers remarkable cost efficiency and near‐zero alloying while maintaining full recyclability. Furthermore, introducing a hierarchical ultrafine‐twinned structure in CP‐Zr has likewise proven effective in simultaneously enhancing both strength and fracture toughness. Therefore, these results establish that pre‐engineered twin networks activating numerous <c+a> dislocations represent a general and sustainable strategy for improving the strength and damage tolerance of HCP metals.

## 4 Experimental Section/Methods

### 4.1 Sample Preparation

Hierarchical ultrafine‐twinned Ti (UFT‐Ti) was fabricated via alternating directional rolling at 77 K (the sample was immersed in liquid nitrogen before each rolling pass, which lasted about 10 min), respectively, through the following protocol. First, the commercial‐purity Ti (CP‐Ti) sample (45 mm length × 18 mm width × 18 mm thickness) underwent ∼10 passes of unidirectional rolling at 77 K, with a thickness reduction of ∼1% each pass, resulting in a total strain of ∼10% along the normal direction (ND). Second, the sample was rotated 90°, followed by an additional 10 passes of rolling at 77 K, with a total thickness reduction of ∼10% (1% each pass) along the original transverse direction (TD). Third, a 450°C/1 h annealing treatment was carried out for residual stress relief. The above processes were then repeated once more to achieve the final sample dimensions of 60 mm (length) × 15 mm (thickness) × 15 mm (width). The high‐density dislocation Ti (HDD‐Ti) was produced using the same alternating directional multi‐pass rolling technique as UFT‐Ti, but all rolling processes were conducted at RT. Its initial size, stress‐relief annealing conditions, and final size were consistent with those of UFT‐Ti, as shown in Figure S1. The annealing was conducted under ultrahigh vacuum (<1 × 10^−4^ Pa) to suppress the uptake of oxygen, nitrogen and hydrogen. During each pass of the rolling process, the roll speed is 17 r/min (line speed 8 m/min), with no lubrication treatment applied to the sample surface. After rolling, the uniform distribution of the microstructure on the RD plane confirms the deformation uniformity during the multi‐pass alternating direction rolling process.

Material compositions (Table S1) confirm nearly identical impurity levels—particularly oxygen (O)—across all three Ti types, with oxygen content quantified using LECO ONH836 Oxygen/Nitrogen/Hydrogen Elemental Analyzer. The hydrogen content in CP‐Ti is measured to be <0.001 wt.%. Furthermore, hydrogen incorporation into the bulk Ti is highly unlikely during rolling processes at both LNT and RT, as well as during subsequent stress‐relief annealing under high vacuum conditions. In addition, at least 1 mm of surface layer was removed to limit surface contamination following rolling and annealing for the preparation of SENB specimens. Starting with CP‐Zr having an average grain size of ∼30 µm, a hierarchical ultrafine‐twinned Zr (UFT‐Zr) sample with twin thickness of ∼0.8 µm was fabricated using the same alternating directional rolling process at 77 K. The composition of CP‐Zr is provided in Table S2. The concentrations of impurities, including C, N, H, and O, are very low, and neither the 77 K rolling nor the subsequent high‐vacuum stress‐relief annealing is expected to increase the impurity levels in UFT‐Zr.

### 4.2 Mechanical Properties Test

Uniaxial tensile tests of CP‐Ti, HDD‐Ti, and UFT‐Ti utilized dog‐bone specimens (12 mm gauge length, 3.0 mm × 2.0 mm gauge cross‐section) wire‐cut from processed sheets via electrical discharge machining (EDM). Engineering strain was measured using a 10‐mm extensometer during room‐temperature tensile testing performed on a 10 kN SANS CMT4104 testing machine at a strain rate of 1.0 × 10^−3^ s^−1^, with the tensile axis parallel to the rolling direction (RD) in the second round of the alternating directional rolling. Triplicate testing ensured reproducibility for each type of Ti samples.

To evaluate fracture toughness, single‐edge notched bend (SENB) specimens (thickness *B* = 6 mm, width *W* = 12 mm, total length *L* = 54 mm) were fabricated from CP‐Ti, HDD‐Ti, and UFT‐Ti samples (Figure S2), with notches (depth = 4.5 mm, root radius ∼100 µm) machined into all specimens; pre‐test preparation included grinding and polishing of the SENB samples.

Fracture toughness tests were conducted in accordance with ASTM Standard E1820 with SENB samples fatigue pre‐cracked to a total crack length *a_0_* = 6.0 mm (0.5 *W*) using a 50 kN high‐frequency fatigue tester (QBG‐50, CCQB) under load‐controlled compression‐compression cycling (frequency = 90 Hz, load ratio is 0.1); subsequent side grooves (thickness reduction = 0.2*B*) were machined on both surfaces to stabilize crack propagation [24]. Three‐point bending tests were performed on a SANS CMT4104 testing machine (span *S* = 48 mm) under displacement control (0.5 mm/min), with crack mouth opening displacement (*d*) monitored via a 5‐mm clip‐on gauge. Fracture toughness values were derived via nonlinear elastic‐fracture mechanics, and *J*‐integral *R*‐curves (*∆a*) were generated through ASTM E1820 (Text S1) [24]. The provisional toughness *J_Q_* is determined as the intersection of the *R*‐curve and the 0.2 mm offset line with a slope of 2σ_0_, where σ_0_ =  1/2(σ_*y*_ + σ_*uts*_) is the effective yield stress, σ_*y*_ is the yield strength, and σ_*uts*_ is the ultimate tensile strength. For *J_Q_* to be considered as a size‐independent fracture toughness (J_IC_) value, the validity requirements for plane‐strain conditions must be satisfied, i.e., *b_0_, B* > 10*J_Q_*/σ_0_, where *b_0_* and *B* are the initial ligament length and specimen thickness, respectively. In other words, the provisional toughness *J_Q_* must be lower than the maximum *J*‐integral (*J_max_* = *b_0_* σ_0_/10). The corresponding *K*‐based fracture toughness values are then calculated using the standard mode‐Ι *J‐K* equivalence relationship: $K_{JIc}={\left(E^{\prime }\cdot J_{Ic}\right)}^{1/2}$.

As shown in Table S3, the effective yield stress σ_0_ is 439.5, 554.3, and 659.2 MPa for CP‐Ti, HDD‐Ti, and UFT‐Ti, respectively. Correspondingly, the calculated 10*J_Q_*/σ_0_ values for CP‐Ti, HDD‐Ti, and UFT‐Ti are 1.7, 1.6 and 4.4 mm, respectively (Table S3). In addition, a provisional crack‐growth fracture toughness (*K_SS_*) was calculated from the crack‐growth *J*‐integral (*J_SS_*) evaluated from the *J*‐*R* curves at *Δa* ∼1.5 mm (Table S3). All *J_Q_* and *K_Q_* of the CP‐Ti, HDD‐Ti, and UFT‐Ti met the specimen size requirements for both *J*‐field dominance (*J_Q_* <  *J_max_*) and plane‐strain conditions (*b_0_*, *B* > 10*J_Q_*/σ_0_) (Table S3), and therefore can be regarded as ASTM‐valid size‐independent fracture toughness of *J_Ic_* and *K_JIc_*.

The tensile properties and fracture toughness of CP‐Zr and UFT‐Zr were also tested based on the above method. The dimensions of all Zr and Ti samples are consistent. The *J_Q_* and *K_Q_* values for commercial‐purity Zr (CP‐Zr) and hierarchical ultrafine‐twinned Zr (UFT‐Zr) were also confirmed to be ASTM‐valid, size‐independent fracture toughness of *J_Ic_* and *K_JIc_*, as summarized in Table S5.

### 4.3 Fractographic Characterization

To investigate crack propagation behavior and deformation mechanisms near crack tips of CP‐Ti, HDD‐Ti, and UFT‐Ti, the SENB samples were ground and polished to 0.05 µm surface roughness, with crack tip deformation morphologies and crack profiles characterized using a Hitachi SU6600 SEM in secondary electron (SE) mode at 15 kV accelerating voltage.

To clarify toughening mechanisms under plane‐strain conditions, side‐grooved SENB samples were test‐interrupted and mid‐thickness sliced during fracture toughness evaluation, with interior surfaces imaged in a Hitachi SU6600 SEM (15 kV). Samples were ground and electrochemically polished (10 vol% HClO_4_ + 90 vol% CH_3_OH, 30 V/−50°C/30 s) before characterization. EBSD scans (Zeiss Gemini 500 SEM, 15 kV, step = 0.08–0.4 µm) captured crack profiles and crack tips, with AztecCrystal software‐generated KAM maps (5 × 5 kernel, 5° max angle, first‐nearest‐neighbor) and GND maps (<a> and <c+a> Burgers vectors) showing strain distributions. SENB samples were further etched (2 vol% HF + 5 vol% HNO_3_ + 43 vol% H_2_O) to highlight crack morphologies. A JEOL 2100F FEG high‐resolution TEM (200 kV, double‐tilt holder) was used to investigate the twin and dislocation structures in pre‐crack and post‐propagation crack‐tip regions, complemented by JEM‐ARM200F‐based HAADF‐STEM for atomic‐scale characterization.

To analyze crack‐tip dislocation structures under plane‐strain conditions, SENB specimens with crack extension of Δ*a* ≈ 1 mm were prepared. Thin mid‐thickness‐plane foils were sectioned via EDM, mechanically ground to ∼50 µm, and punched into 3 mm discs containing crack tips. Discs were dimpled centrally and twin‐jet electropolished at −50°C (30 V) using 10 vol% perchloric acid and 90 vol% methanol electrolytes. For crack‐tip thinning, the front region was pre‐ground to a thickness of ∼30 µm using a Model 200 dimpler, then foil‐shielded during electropolishing to prevent light transmission. Final thin zones within the plastic zone were obtained, with dimpling‐induced deformation excluded by comparing samples before and after dimpling. Standard **g·b** analysis (**g** = diffraction vector, **b** = Burgers vector) was conducted under two‐beam conditions.

### 4.4 Statistical Analysis

Tensile properties, including yield strength (*σ_y_*), ultimate tensile strength (*σ_uts_*), and elongation to fracture (*ε_f_*), were determined from the tensile engineering stress–strain curves. The average values and standard deviations of these tensile properties were calculated from three dog‐bone specimens and summarized in Tables S3 and S5. Mean and standard deviations of fracture toughness were also calculated from three SENB specimens and displayed in Tables S3 and S5. The twin boundary density (Figure S13) and KAM values (Figure 3f) before and after crack propagation were calculated as the average value with standard deviations from three samples.

## Author Contributions

W.Z.H. conceived the project and supervised the research. X.W.Z. performed the mechanical tests and microstructural characterizations under the guidance of W.Z.H., X.W.Z., E.M. and W.Z.H. wrote the paper. All authors analyzed the data and contributed to the discussion of the results.

## Conflicts of Interest

The authors declare no conflict of interest.

## Supporting information




**Supporting File**: advs75805‐sup‐0001‐SuppMat.docx.

## Data Availability

The data that support the findings of this study are available in the supplementary material of this article.
