# Supplementary material for: Hierarchical Twin Networks Enable Exceptional Strength and Fracture Toughness in Titanium
Source: Adv Sci (Weinh). 2026 May 21;13(46):e75805. doi: 10.1002/advs.75805 (PMC13335867; doi:10.1002/advs.75805)
Supplement: Supplementary file 1 [file ADVS-13-e75805-s001.docx]

Supporting Information

**Hierarchical twin networks enable exceptional strength and fracture toughness in titanium**

*Xiao-Wei Zou*, *Ting Zhu*, *En Ma, and Wei-Zhong Han**

*Corresponding to [w.z.han@cityu.edu.hk](mailto:w.z.han@cityu.edu.hk)

Supplementary Text

Figures S1-S24

Tables S1-S6

References

Supplementary Text

**Fracture toughness testing**

To evaluate fracture toughness, single-edge notched bend (SENB) specimens (thickness *B* = 6 mm, width *W* = 12 mm, total length *L* = 54 mm) were fabricated from commercial-purity Ti (CP-Ti), high-density dislocation Ti (HDD-Ti), and hierarchical ultrafine-twinned Ti (UFT-Ti) (Figure S2), with notches (depth = 4.5 mm, root radius ∼ 100 μm) machined into all specimens; pre-test preparation included grinding and polishing of the SENB samples.

Fracture toughness tests were conducted in accordance with ASTM Standard E1820 with SENB samples fatigue pre-cracked to a total crack length *a_0_* = 6.0 mm (0.5 *W*) using a 50 kN high-frequency fatigue tester (QBG-50, CCQB) under load-controlled compression-compression cycling (frequency = 90 Hz, load ratio is 0.1); subsequent side grooves (thickness reduction = 0.2*B*) were machined on both surfaces to stabilize crack propagation.^[1]^ Three-point bending tests were performed on a SANS CMT4104 testing machine (span *S* = 48 mm) under displacement control (0.5 mm/min), with crack mouth opening displacement (*d*) monitored via a 5-mm clip-on gauge. Fracture toughness values were derived via nonlinear elastic-fracture mechanical method, while *J*-integral *R*-curves (*∆a*) were generated through ASTM E1820. ^[1]^

During compression loading, the SENB specimens were periodically unloaded to ~20% of peak load to record the elastic compliance by using clip-on gauge. The crack length, *a_i_* was calculated from elastic unloading compliance:

$\frac{a_{i}}{W}=0.999748 -3.9504u+2.9821u^{2}-3.21408u^{3}+51.51564u^{4}-113.031u^{5},$ (1)

$u=\frac{1}{{(B_{e}EC_{i})}^{1/2} +1},$	(2)

$C_{i}={\Delta d_{i}}/{\Delta P_{i}},$	(3)

Where $B_{e}=B-\left( B-B_{N} \right)^{2}/B$, $B_{N}$ is the net side-grooved thickness (~4.8 mm), $C_{i}$ is the experimental compliance, $d_{i}$ is the crack mouth opening displacement at notched edge and $P_{i}$ is the applied load prior to the partial unloading. For each crack length $a_{i}$, $J_{i}$ value was calculated as the sum of elastic $J_{e(i)}$ and plastic $J_{pl(i)}$ components:

$J_{i}=J_{e(i)}+J_{pl(i)}=\frac{K_{i}^{2}}{E^{'}}+J_{pl(i)},$	(4)

where $E^{'}=E$, the Young’s modulus in plane stress and $E^{'}=E/(1-v^{2})$ in plane strain, $v$ is Poisson’s ratio. For pure Ti, *E* and $v$ are 108 GPa and 0.33, respectively.^[2]^ For pure Zr, *E* and $v$ are 95 GPa and 0.33, respectively.^[3]^ *K_i_* is the linear elastic stress intensity corresponding to each data point on the load-crack mouth opening displacement curve:

$K_{i}=\frac{P_{i}*S}{{(BB_{N})}^{1/2}*W^{3/2}}\cdot f(\frac{a_{i}}{W}),$	(5)

where $f(\frac{a_{i}}{W})$ is a geometry-dependent function of the ratio of crack length, *a_i_*, to width, *W*, as listed in the ASTM Standard.^[1]^ The plastic component of *J_i_* can be calculated from the following expression:

$J_{pl(i)}=\left[ J_{pl\left( i-1 \right)}+\left( \frac{\eta_{pl\left( i-1 \right)}}{b_{\left( i-1 \right)}} \right)\left( \frac{A_{pl\left( i \right)}-A_{pl\left( i-1 \right)}}{B_{N}} \right) \right]\times\left[ 1-\gamma_{\left( i-1 \right)}\left( \frac{a_{i}-a_{i-1}}{b_{i-1}} \right) \right],$	(6)

where$\eta_{pl\left( i-1 \right)}=3.667-2.199\frac{a_{\left( i-1 \right)}}{W}+0.437{(\frac{a_{\left( i-1 \right)}}{W})}^{2}$ and $\gamma_{\left( i-1 \right)}=0.131+2.131\frac{a_{\left( i-1 \right)}}{W}-1.465{(\frac{a_{\left( i-1 \right)}}{W})}^{2}$.$A_{pl\left( i \right)}$ is the (plastic work) area under the load-crack mouth opening displacement curve, and $b_{i}=W-a_{i}$ is the ligament length. Using Eqs. (1) to (6), the *J*-∆*a* resistance curve can be constructed by calculating the *J_i_*-integral corresponding to the crack extension ∆$a_{i}$ (the difference of the crack length $a_{i}$ and the initial crack length, $a_{0}$).

The provisional toughness $J_{Q}$ is determined as the intersection of the *R*-curve and the 0.2 mm offset line with a slope of 2$\sigma_{0}$, where $\sigma_{0}=1/2(\sigma_{y}+\sigma_{uts})$ is the effective yield stress, $\sigma_{y}$ is the yield strength, and $\sigma_{uts}$ is the ultimate tensile strength. For $J_{Q}$ to be considered as a size-independent fracture toughness ($J_{Ic}$) value, the validity requirements for plane-strain conditions must be satisfied, i.e., *b_0_, B* > 10$J_{Q}/\sigma_{0}$, where *b_0_* and *B* are the initial ligament length and specimen thickness, respectively. In other words, the provisional toughness *J_Q_* must be lower than the maximum *J*-integral (*J_max_* = *b_0_*$\sigma_{0}$/10). The corresponding *K*-based fracture toughness values are then calculated using the standard mode-Ι *J-K* equivalence relationship: $K_{JIc}={(E^{'}\cdot J_{Ic})}^{1/2}$.

As shown in Table S3, the effective yield stress $\sigma_{0}$ is 439.5 MPa, 554.3 MPa, and 659.2 MPa for CP-Ti, HDD-Ti, and UFT-Ti, respectively. Correspondingly, the calculated 10$J_{Q}/\sigma_{0}$ values for CP-Ti, HDD-Ti, and UFT-Ti are 1.7 mm, 1.6 mm and 4.4 mm, respectively (Table S3). In addition, a provisional crack-growth fracture toughness (*K_SS_*) was calculated from the crack-growth *J*-integral (*J_SS_*) evaluated from the *J*-*R* curves at *Δa* ~1.5 mm (Table S3). All $J_{Q}$ and $K_{Q}$ of the CP-Ti, HDD-Ti, and UFT-Ti met the specimen size requirements for both *J*-field dominance ($J_{Q}< J_{max}$) and plane-strain conditions (*b_0_*, *B* > 10$J_{Q}/\sigma_{0}$) (Table S3), and therefore can be regarded as ASTM-valid size-independent fracture toughness of *J_Ic_* and *K_JIc_*. In addition, the $J_{Q}$ and $K_{Q}$ values for commercial-purity Zr (CP-Zr) and hierarchical ultrafine-twinned Zr (UFT-Zr) were also tested and confirmed to be ASTM-valid, size-independent fracture toughness of *J_Ic_* and *K_JIc_*, as summarized in Table S5.

**Evolution of deformation mode**

Tensile loading induced distinct plastic deformation patterns (Figure S5): CP-Ti and HDD-Ti showed sparse twin activation in uniform deformation zones (Figure S5a,d) versus intensive twinning near fracture areas (Figure S5b,c,e,f), while UFT-Ti exhibited dense twins spanning both uniform/fracture regions (Figure S5g-i). Interestingly, tensile-aligned twin elongation near fracture sites (Figure S5h,i) demonstrated pre-existing high-density twins' capacity for substantial plastic accommodation—consistent with fully ductile fracture surfaces containing abundant dimples across all three Ti samples (Figure S6). This deformation twin-mediated plasticity continuum fundamentally differentiates UFT-Ti's enhanced ductility from CP-Ti and HDD-Ti counterparts.

Toughening mechanism differentiation in CP-Ti, HDD-Ti and UFT-Ti was established through integrated analysis of fracture mode evolution (Figure S7) coupled with pre-polished surface (Figure S8) and side-grooved SENB mid-thickness plane (Figure S9) deformation features. Fracture surface quantification (Figure S7a,c,e) demonstrated cross-sectional shrinkage dimensions during crack propagation being thickness-negligible (~4%) across all three Ti samples, validating plane-strain dominance—though UFT-Ti's relatively enlarged necking reflected enhanced crack-tip plasticity. SEM fractography revealed CP-Ti's semi-brittle propagation zones dominated by cleavage facets with sparse dimples (Figure S7b), and HDD-Ti showed analogous morphology with moderate dimple density increase (Figure S7d). Strikingly, UFT-Ti exhibited fully ductile fracture characterized by uniform high-density dimple distribution (Figure S7f), providing direct microstructural evidence of its intense plastic deformation during crack advancement under plane-strain constraints.

Crack-tip plastic deformation capacity was quantified via in-situ digital image correlation (DIC) (Ncorr 2D-DIC MATLAB software), revealing butterfly-shaped surface plastic zones at crack initiation in all three Ti SENB specimens (Figure S8a,e,i). Both CP-Ti and HDD-Ti exhibited reduced plastic zone dimensions and strain magnitudes versus UFT-Ti's expanded zone with elevated strain—providing direct evidence of crack-tip deformation capacity disparity. SEM surface analysis at Δ*a* ~2 mm showed CP-Ti and HDD-Ti with narrow cracks, sparse and non-uniform deformation (with limited slip bands in Figure S8s,d,g,h), contrasting UFT-Ti's broad crack (Figure S8j) accompanied by uniform plasticity and dense slip band activation (Figure S8k,l). This intensified homogeneous plastic deformation under plane-stress conditions constitutes the intrinsic crack-arresting mechanism of UFT-Ti through energy dissipation maximization.

Under mid-thickness plane-strain conditions, CP-Ti exhibited minimal deformation twinning during crack propagation (Figure S9a-c), with crack-adjacent grains retaining equiaxed structures indicative of limited plasticity. Both CP-Ti and HDD-Ti developed flat and straight crack paths with small *CTOD* (Figure S9b,e). Conversely, UFT-Ti demonstrated substantially increased *CTOD* and tortuous crack trajectories (Figure S9g,h), where high-density twins induced frequent crack deflection—a micro-mechanism critically enhancing crack-growth toughness of UFT-Ti through energy dissipation diversification.

KAM and GND density maps (Figures S10 and S14) substantiated that the hierarchical ultrafine twins of UFT-Ti intensified dislocation activity during crack propagation, with expanded high-KAM and GND zones along crack profiles—particularly <a> and <c+a> slip systems in Figure S14h,i—evidencing enhanced plasticity. Conversely, CP-Ti and HDD-Ti confined high KAM and GND density to grain-boundary vicinities near crack profiles (Figure S14), demonstrating limited and non-uniform plasticity at the crack tip. This localized plasticity dissipation in CP-Ti and HDD-Ti fundamentally constrained crack initiation and propagation resistance compared to twin-engineered delocalization in UFT-Ti. In addition, no significant changes in twin boundary density, twin types, or twin sizes are observed before and after crack propagation (Figures S12, S13), further confirming that dislocation slip, rather than twinning or detwinning, is the primary deformation mechanism at room temperature under the triaxial stress conditions at the crack tip.

TEM analysis of CP-Ti revealed negligible pre-existing <a> or <c+a> dislocations in initial grains (Figure S15). Post-cracking, dense <a> but sparse <c+a> dislocations activated within the crack-tip plastic zone were confirmed by dislocation visibility transition under **g** = 0$\bar{1}11$→**g** = 0002 (Figure S15d-f), demonstrating limited activation of <c+a> slip under crack-tip stress. In contrast, HDD-Ti initially exhibited uniformly distributed <c+a> dislocations (Figure S16a-d), with many aligned parallel to pyramidal-basal plane intersections (Figure S16c,d) at characteristic angles (105° or 90° between Burgers vectors and dislocation lines), conferring dominant edge components with low mobility.^[4-8]^. Post-crack propagation maintained near-constant <c+a> dislocation density (Figure S16e-h), their persistent edge-character alignment evidencing restricted self-multiplication due to screw versus edge component mobility disparity (see details in the next section).^[9,10]^

Initial UFT-Ti exhibited abundant dislocations with <a>-type density exceeding <c+a> types, as verified by **g** = 0002 and **g** = $\bar{\text{2}}$110 two-beam conditions (Figure S17a,b). <c+a> dislocations at twin boundaries displayed characteristic "bowing-out" morphologies, suggesting twin boundaries act as dislocation sources. Notably, basal stacking fault-contacting <c+a> dislocations (Figure S17d,e) provided direct evidence for <c+a> edge dislocation dissociation along basal planes.^[7]^ Post-crack propagation (Δa ~ 1 mm) activated substantially more <c+a> dislocations, with intensified bowing-out configurations observed at twin boundaries (Figure S17g-k). Twin-boundary-mediated emission events, including bilateral dislocation nucleation (Figure S17k), demonstrate the capacity of hierarchical ultra-dense twin network to generate <c+a> dislocations propagating into both matrix and twin domains, thereby enhancing crack-tip plastic uniformity in UFT-Ti.

TEM characterization of twin-boundary-associated dislocations revealed: at {11$\bar{\text{2}}$2} (C1) twin boundaries (Figure S19a-c), matrix-side <a> dislocations (yellow arrows) showed visibility transition under **g** = 0002→**g** = 11$\bar{\text{2}}$0, while twin-side <c+a> dislocations (red arrows) remained visible under **g** = 0002. Direct matrix-twin dislocation connectivity was observed via <a> to <c+a> linkage across C1 boundaries (boxed region, Figure S19c). Similar interactions at {11$\bar{\text{2}}$1} (T2) boundaries (Figure 4k-m) demonstrated exclusive <c+a> visibility under **g** = 0002 (red arrows, Figure 4k), with <a> dislocations emerging under matrix and twin-specific **g** = 11$\bar{\text{2}}$0 conditions (yellow arrows, Figure 4l,m). Cross-boundary <a> and <c+a> dislocation networks formed at T2 interfaces (boxed regions, Figure 4m), confirming bidirectional dislocation transformation mechanisms, which can be a source for <c+a> dislocations.

For HCP Ti, <a> dislocations exhibit much higher activation propensity than <c+a> dislocations, leading to crack-tip <a> dislocation accumulation in UFT-Ti that interacts with pre-existing twin boundaries. When the initial <a> dislocation (from matrix or twin) impinges on a twin boundary, the interaction follows <a>→1/2<c+a> + 1/2b_t_, generating 1/2<c+a> dislocations and 1/2b_t_ at twin boundaries (Figure S19a,e). Subsequent interaction of a second <a> dislocation (identical Burgers vector) produces another 1/2<c+a> dislocation and 1/2b_t_ dislocation, which combines with the pre-existing 1/2<c+a> to form a perfect <c+a> dislocation alongside b_t_ twinning dislocations (Figure S19f,g). The localized accumulation of b_t_ twinning dislocations likely induces twin-boundary step formation, as experimentally observed at multiple twin boundaries (Figure 4h-j). Collectively, these <a>-twin boundary interactions create pathways for <c+a> dislocation nucleation.

Notably, after a large number of <c+a> dislocations nucleate at the twin boundary ahead of the crack tip, when these dislocations encounter an adjacent twin boundary during slip, they are not only hindered by the boundary, but some <c+a> dislocations on specific slip planes may also be transmitted across the coherent regions of the twin boundary (Figure S20). This slip transmission mechanism can reduce the stress concentration at the boundaries and further promote the energy dissipation at the crack tip.

In summary, twinning-stimulated <c+a> dislocation activation at crack tips induced uniform yet severe plastic deformation, enabling record-high fracture resistance in UFT-Ti.

**Self-multiplication efficiency of <c+a> dislocations**

In general, the Frank-Read type dislocation source is the primary model for dislocation multiplication in recrystallized coarse-grained metals, requiring the cooperative propagation of screw and edge dislocations. For example, an ideal regeneratable Frank-Read dislocation source is easily formed when screw and edge dislocations have similar mobilities,^[9,10]^ such as when *v_s_* = *v_e_*, where *v_s_* and *v_e_* represent the velocities of edge and screw dislocations, respectively, as shown in Figure S21a. In practice, however, edge and screw dislocations typically exhibit different mobilities due to their distinct core structures, which limits the operational efficiency of dislocation sources.^[3,9,10]^

As illustrated in Figure S21b, the bowing edge dislocation initially forms a half-loop with radius *r*. When edge dislocations have much lower mobility than screw dislocations (*v_e_* ≪ *v_s_*), in the extreme case where *v_e_* = 0, the half-loop can only expand forward by a distance *x*, forming a half ellipse, while side glide of the dislocation is impossible due to the immobility of the edge dislocation. Consequently, such a dislocation source behaves as a disposable source, with the screw segments eventually disappearing at the free surface or grain boundaries, leaving behind two long, straight edge dislocations (Figure S21b). The light blue region represents the area (*A_screw_*) swept by the screw dislocation in this case.

As *v_e_* increases, the bowing of the half-loop forms a purple half ellipse, as shown in Figure S21c, exhibiting both a forward glide distance of *x* and a side glide distance of *y*. The purple area represents the region (*A_edge_*) swept by the edge dislocation. Once the area swept by edge dislocations exceeds that swept by screw dislocations, the bowing segment can go around and return, thus evolving into a Frank-Read dislocation source.^[3,9,10]^ Therefore, the equality of the areas swept by edge and screw dislocations (*A_edge_* = *A_screw_*) defines the critical condition for the formation of a Frank-Read dislocation source. From their geometrical relation, it can be derived that:

$A_{screw}=\frac{\pi(x+r)r}{2}-\frac{\pi r^{2}}{2}$,	(7)

$A_{edge}=\frac{\pi(x+r)(y+r)}{2}-\frac{\pi(x+r)r}{2}$,	(8)

If *A_screw_* = *A_edge_*, we can obtain:

$\frac{y}{x}=\frac{r}{x+r}$,	(9)

The mobility ratio of screw versus edge dislocations (*α*) is related to the glide distances of screw and edge components, such as:

$\alpha=\frac{v_{e}}{v_{s}}=\frac{y}{x}=\frac{r}{x+r}$,	(10)

Here, *α* is related to the geometry of dislocation bowing, reflecting the efficiency of dislocation sources. We propose that the efficient operation of the Frank-Read source requires 0 < *x* < *r*, where *r* is the dislocation source radius. If *x* > *r*, the dislocation source is a low-efficiency disposable one. Therefore, *x* = *r* (where *α* = 0.5) is a critical requirement for effective operation of the Frank-Read source, as shown in Figure S21d,e. The value of *α* represents the efficiency of the dislocation source, determining the capacity for dislocation multiplication.

For HCP metals such as Ti, Zr, and Mg, the dissociation of <c+a> edge component on the basal plane and the formation of a three-dimensional dislocation core result in its glide velocity of being only 10% that of screw component.^[4-8]^ This leads to an *α* value for <c+a> dislocations that is far below 0.5, indicating that the <c+a> dislocation source in HCP-Ti is low-efficiency and non-renewable. Consequently, <c+a> dislocations at the crack tip are unable to multiply effectively, which limits the fracture toughness of Ti.

**Evolution of microstructure and mechanical properties of Zr**

To investigate the impact of high-density twin boundaries on the mechanical properties of other HCP metals, we fabricated a UFT-Zr with a hierarchical ultrafine-twinned structure using the same alternating directional rolling process employed for UFT-Ti, starting with a CP-Zr. The initial CP-Zr exhibits an equiaxed grain structure with an average grain size of ~31 μm, containing no detectable deformation twins or dislocations (Figure S22a,b). Following alternating directional rolling at 77 K, numerous deformation twins—mainly sub-micron and nano-scale {10$\bar{\text{1}}$2} (T1) and {11$\bar{\text{2}}$1} (T2) variants—were observed within slightly elongated grains in the UFT-Zr (Figure S22c-f). The average twin thickness of UFT-Zr is ~0.8 μm, resulting in a refined average grain size of ~3.4 μm.

Uniaxial tensile tests and nonlinear elastic fracture mechanics tests were conducted at RT to compare the mechanical properties of CP-Zr and UFT-Zr. Due to the strengthening effect of twin boundaries, the yield strength of UFT-Zr increases to 411 MPa (Figure S23a), nearly 100% higher than that of CP-Ti (210 MPa). Notably, the ultrafine twin boundary network also enhanced the fracture toughness of Zr (Figure S23b). UFT-Zr achieves a crack initiation toughness *J_Ic_* of ~130 kJ/m^2^ (Table S5), which is three times that of CP-Zr (45 kJ/m^2^). Using *J*-*K* equivalence, the *K*-based fracture toughness of UFT-Zr is found to be 118 MPa·m^1/2^, nearly 71% higher than that of CP-Ti (69 MPa·m^1/2^). Therefore, similar to HCP-Ti, the introduction of hierarchical ultrafine-twinned structure in HCP-Zr can also overcome the conflict between strength and fracture toughness.^[11]^

To clarify the toughening mechanisms, we systematically investigated the evolution of deformation and fracture morphologies for CP-Zr and UFT-Zr SENB specimens. At crack initiation on the sample surface, the crack-tip plastic zone of UFT-Zr is significantly larger than that of CP-Ti (Figure S24a,e). Moreover, UFT-Zr exhibits higher, rather than limited strain, compared to CP-Zr. SEM analysis of the pre-polished specimens at crack extension Δ*a* ≈ 2 mm (Figure S24b-d,f-h) further confirms this difference—UFT-Zr shows severe and homogeneously distributed plasticity events, whereas CP-Zr experiences localized and slight deformation. During crack propagation at the mid-thickness surface, under plane-strain conditions, the crack-tip opening displacement (*CTOD*) of UFT-Zr is nearly twice that of CP-Zr at the same crack extension of Δ*a* ≈ 2 mm. This higher *CTOD* value further confirms the enhanced fracture resistance of UFT-Zr. The ultrafine twin boundary network also influenced the fracture mode under crack-tip triaxial stress states. CP-Zr displays typical semi-brittle fracture characteristics, dominated by cleavage facets with sparse dimples (Figure S24k), consistent with their limited crack-tip plasticity. In contrast, UFT-Zr undergoes a completely ductile fracture with uniformly distributed high-density dimples (Figure S24l). This change in fracture mode can be attributed to the twin boundary network facilitated crack-tip plasticity, which hindered both the crack initiation and propagation, resulting in the simultaneous enhancement of strength and fracture toughness in HCP-Zr.

Overall, the introduction of ultrafine twin boundary networks is an effective and universal strategy for achieving synergistic strengthening and toughening, while overcoming the inherent fracture toughness limitations of HCP metals (e.g., Ti, Zr, Mg, Zn, Be).

**Supplementary Figures**

**Figure S1.** Illustration of alternating directional rolling and heat-treatment procedures used to fabricate bulk HDD-Ti and UFT-Ti. CP-Ti underwent ~10 passes of rolling, with a thickness reduction totaling ~10% in both the normal direction (ND) and transverse direction (TD), at room temperature (RT) or liquid nitrogen temperature (LNT), followed by relaxation annealing at 450 °C for 1 hour. This process is then repeated once more to produce HDD-Ti and UFT-Ti, respectively.

**Figure S2.** Fracture toughness test of CP-Ti, HDD-Ti and UFT-Ti. a,b) Images and schematic illustrations depicting the single-edge bend testing process. c) Plot of the SENB sample with a thickness of *B* = 6 mm**.**

**Figure S3.** Initial microstructures of CP-Ti, HDD-Ti, and UFT-Ti. a-c) EBSD IPF maps and d-f) SEM images reveal significantly different initial microstructures. g,h) SEM images showing ultra-dense twin networks in UFT-Ti. i) Distribution frequency of twin lamella thickness in UFT-Ti. j-l) Distribution frequency of grain size. m) Average grain size of the three types of Ti.

**Figure S4.** Back stress measurement for CP-Ti, HDD-Ti, and UFT-Ti. a) Loading-unloading-reloading (LUR) tensile stress-strain curves. b) Hysteresis loops. c) Schematic illustration for calculating the back stress. d) Back stress at different strain levels. UFT-Ti exhibited the highest back stress. e-g) TEM bright-field (BF) and dark-field (DF) images of UFT-Ti using **g** = 0002 showing the pile-up of <c+a> dislocations at a twin boundary within either the matrix or the twin. The numerous twin boundaries in UFT-Ti promoted dislocation pile-up, leading to significant twin boundary (i.e., grain size) strengthening, which explains the high back-stress in (d).

**Figure S5.** SEM images of deformation morphologies after uniaxial tensile test for CP-Ti, HDD-Ti, and UFT-Ti. a) A few twins are observed in uniform deformation region of CP-Ti. b,c) More twins are activated near the fracture region. d) Many randomly oriented twins are distributed in uniform deformation region of HDD-Ti. e,f) Most twins are elongated along tensile direction, with some micro-voids formed near the fracture region. g) A much higher density of twins is observed in uniform deformation region of UFT-Ti. h,i) Most twins are elongated near the fracture region, and uniformly dispersed micro-voids nucleate preferentially at twin boundaries.

**Figure S6.** SEM images of fracture surface after uniaxial tensile test for CP-Ti, HDD-Ti, and UFT-Ti. All three Ti samples exhibit fully ductile fracture modes, characterized by numerous dimples. b,d,f) is magnified images of a,c,e), respectively. The average dimple diameters (*d_avg._*) of CP-Ti, HDD-Ti, and UFT-Ti are 2.7, 1.6, and 1.3 μm, respectively.

**Figure S7.** SEM images of fracture surface after SENB test for CP-Ti, HDD-Ti, and UFT-Ti. a,b) Crack propagation surface in CP-Ti is relatively flat, exhibiting typical semi-brittle fracture mode, characterized by numerous flat facets and a few dimples. c,d) HDD-Ti displays a similar semi-brittle fracture morphology, but with more dimples. e,f) UFT-Ti exhibits a completely ductile fracture mode, with a large number of dimples distributed throughout the entire crack propagation region. Images (i) and (ii) show magnified views.

**Figure S8.** Deformation microstructures for CP-Ti, HDD-Ti, and UFT-Ti SENB samples at the pre-polished surface. a) Two-dimensional strain distribution of CP-Ti near the crack tip at crack initiation (∆*a* ≈ 0.2 mm), obtained using the DIC method, shows a small plastic zone accompanied by minimal deformation. b) SEM image reveals a small crack tip opening displacement (*CTOD*). c,d) The enlarged SEM images of (b) show a significant non-uniform deformation morphology, with severe deformation regions featuring numerous slip bands, and slight deformation regions with fewer slip bands, observed near the crack profile. e) Strain distribution map at crack initiation (∆*a* ≈ 0.2 mm) indicates a similar small plastic zone with low strain ahead of the crack tip for HDD-Ti. f) SEM image shows a small *CTOD*. g,h) Enlarged images of (f) show a non-uniform deformation morphology near (g) crack profile and (h) crack tip. Many micro-cracks are formed, as indicated by the yellow arrows in (g). i) UFT-Ti exhibits the largest plastic zone with the most severe deformation ahead of the crack tip at crack initiation (∆*a* ≈ 0.2 mm). j) A much larger *CTOD* is observed in UFT-Ti at the same crack extension (∆*a* ≈ 2 mm, consistent with CP-Ti and HDD-Ti). k) Extensive plastic deformation, without cracking along the twin boundaries. l) Dense slip bands are activated. These deformation modes contribute significant plastic energy dissipation during crack propagation. The stress intensity factor (*K_i_*) at the crack tip for CP-Ti, HDD-Ti, and UFT-Ti are ~ 41, 56, 68 MPa·m^1/2^ at crack initiation (∆*a* ≈ 0.2 mm), and ~ 40, 51, 67 MPa·m^1/2^ at a crack extension of ∆*a* ≈ 2 mm.

**Figure S9.** Deformation microstructures of CP-Ti, HDD-Ti, and UFT-Ti SENB samples at the mid-thickness surface (plane-strain condition). a) A small *CTOD* is observed in CP-Ti at a crack extension of ∆*a* ≈ 2 mm. b,c) Enlarged optical microscope (OM) and SEM images ahead of the crack tip confirm that only a few deformation twins have been activated. d) HDD-Ti also exhibits a relatively small *CTOD*. e,f) Enlarged OM and SEM images reveal that pre-existing twins are present in some grains, while others show difficulty in activating additional twins. g) UFT-Ti displays a significantly larger *CTOD* at the same crack extension. h,i) A much higher density of deformation twins is observed near both the crack profile and crack tip.

**Figure S10.** EBSD images near the crack tip at the mid-thickness surface of CP-Ti, HDD-Ti, and UFT-Ti (∆*a* ≈ 2 mm). a-c) IPF maps reveal a much larger number of twins ahead of the crack tip in UFT-Ti compared to CP-Ti and HDD-Ti. d-f) KAM maps show that UFT-Ti exhibits ultra-dense and uniform plastic deformation at crack tip.

**Figure S11.** EBSD images showing the initial and deformed microstructures near the crack profile at the mid-thickness surface of CP-Ti, HDD-Ti, and UFT-Ti. No significant activation of additional deformation twins is observed near d-f) crack profile, when compared to a-c) the initial structure in all three Ti samples.

**Figure S12.** EBSD images showing the types and distribution of twin boundaries before and after cracking for CP-Ti, HDD-Ti, and UFT-Ti. Compared to a-c) the initial structure, no significant changes are observed in the types and distribution of twin boundaries near d-f) crack profile and g-i) crack tip on the mid-thickness surfaces of the three Ti SENB samples. The crack extension in panels (d-i) for all Ti samples is ~2 mm.

**Figure S13.** Comparison of twin boundary (TB) density before and after cracking. The twin boundary density of the three Ti SENB samples shows only minor changes between the initial state and after a 2 mm crack extension.

**Figure S14.** Comparison of the deformation behavior near the crack profile, in the CP-Ti, HDD-Ti, and UFT-Ti. a1-f1) KAM maps showing the microstructure before (a1-c1) crack propagation and near the (d1-f1) crack profile after the propagation of the crack tip to 2 mm. a2-f2) Geometrically necessary dislocation (GND) maps of <a> type for the (a2-c2) initial and (d2-f2) near-crack microstructures. a3-f3) GND maps of <c+a> type for the (a3-c3) initial and (d3-f3) near-crack microstructures. g-i) Variation in average KAM values, average <a>-type GND density, and average <c+a>-type GND density for the initial and near-crack microstructures. UFT-Ti exhibits a much larger plastic zone compared to CP-Ti and HDD-Ti, as reflected by the high KAM values spread over a much wider region near the crack profile, along with the marked increase in <a>-type and <c+a>-type average GND density after crack propagation.

**Figure S15.** Dislocation structures in CP-Ti. a-c) TEM images of the initial as-received microstructures using **g** = 0$\bar{\text{1}}$11, **g** = 0$\bar{\text{1}}$10, and **g** = 0002, respectively, where no <a> or <c+a> dislocations are observed. d-f) TEM images near the crack tip (within the plastic zone) at the crack extension of ∆*a* ≈ 1 mm. A small number of <c+a> dislocations are activated, as observed using **g** = 0002 in (f). Numerous <a> dislocations are observed using **g** = 0$\bar{\text{1}}11$ in (d,e). Panel (e) is the corresponding dark-field image of (d).

**Figure S16.** TEM images of dislocation structures in HDD-Ti. a,b) DF and BF images of the as-rolled microstructures under **g** = 01$\bar{1}$0 and **g** = 0002 showing a large number of <a> and <c+a> dislocations. c,d) The <c+a> dislocations exhibit a large edge component, with a character angle of 105° (slipping on the first-order pyramidal plane) or 90° (slipping on the second-order pyramidal plane), as verified by the inset in (d). e-h) BF images near the crack tip (within the plastic zone) at crack extension of ∆*a* ≈ 1 mm also show numerous <c+a> dislocations under **g** = 0002. The density of <c+a> dislocations does not increase significantly compared to the initial state, and a large number of <c+a>-edge components remain, implying that these pre-existing <c+a> dislocations are difficult to self-multiply during crack propagation.

**Figure S17.** TEM images of dislocation structures in UFT-Ti. a-f) BF and DF images of the initial structures. Two-beam condition analysis using (a) **g** = 0002 and (b) **g** = $\bar{\text{2}}$110 shows <c+a> dislocations and <a> dislocations inside the twin. Some <c+a> dislocations are observed to be emitted from the {11$\bar{\text{2}}$1} twin boundary, as revealed by their bowing-out shape in (a). (c,d) BF images show that <c+a> dislocations can be emitted from the {10$\bar{\text{1}}$2} twin boundary. e) Enlarged image of (d) shows some stacking faults (SF) along the basal pane, as indicated by red arrows. <c+a> dislocations can be emitted from (f) {11$\bar{\text{2}}$2} twin boundary. g-k) BF and DF images near crack tip (within the plastic zone) at the crack extension of ∆*a* ≈ 1 mm using **g** = 0002. A much larger number of <c+a> dislocations have been activated, with numerous <c+a> dislocations emitted from the (g-i) {10$\bar{\text{1}}$2}, (j) {11$\bar{\text{2}}$2}, and (k) {11$\bar{\text{2}}$1} twin boundaries. <c+a> dislocations also exhibit a long edge component, as indicated by the inset in (h). (k) A twin boundary can emit <c+a> dislocations into both the matrix and the twin.

**Figure S18.** Measured c/a ratio in initial UFT-Ti. a) High-angle annular dark-field STEM (HAADF-STEM) image of a {10$\bar{\text{1}}$2} twin boundary. b) HAADF-STEM image of a {11$\bar{\text{2}}$1} twin boundary. The c/a ratio in the twin is higher than that in the matrix for both {10$\bar{\text{1}}$2} and {11$\bar{\text{2}}$1} twins.

**Figure S19.** Interaction of <a> dislocation with twin boundary in UFT-Ti. a) No <c+a> dislocations are observed in the matrix using **g** = 0002_M_. The inset indicates that it is a {11$\bar{\text{2}}$2} (C1) compression twin. b) A large number of <a> dislocations are present in the matrix using **g** = 11$\bar{\text{2}}$0_M_, and a few <c+a> dislocations can be observed in the twin using **g** = 0002_C1_. c) Enlarged DF-TEM image of (b) shows that two <a> dislocations (indicated by yellow arrows) appear to interact with the twin boundary, generating a <c+a> dislocation (indicated by a red arrow). d-g) Schematic illustrations depict the successive incorporation and transformation of two <a> dislocations at a twin boundary. Panel (g) presents the detailed configuration of the dislocation junction, as highlighted by the dashed circle in (f). “**b_t_**” in (e-g) denotes a twinning dislocation on the twin plane.

**Figure S20.** TEM images showing the slip transmission through the twin boundary for <c+a> dislocations in HTS-Ti. a-c) The slip transmission of <c+a> dislocations across the twin boundary is demonstrated. <c+a> dislocations on both sides of the twin boundary are observed in (b) the matrix and (c) the twin using g = 0002, respectively. d-f) The slip of <c+a> dislocations across the twin boundary is observed in multiple regions, as depicted by the red dashed lines.

**Figure S21.** Efficiency of <c+a> dislocation source. The efficiency of the <c+a> dislocation source is defined as the mobility ratio of edge to screw components (*α* = *v_e_*/*v_s_*).^[9,10]^ Initially, a screw dislocation bows out and forms a half-loop of radius *r*, which then evolves into dislocation sources as both edge and screw dislocations continue to glide. a) An efficient Frank-Read dislocation source with *α* = 1. b) Disposal dislocation source with *α* = 0. c) A low-efficiency dislocation source (*α* < 0.5). d) A dislocation source with moderate efficiency (*α* = 0.5). e) A high-efficiency dislocation source (*α* > 0.5). In the figure, *x* and *y* represent the glide distances of screw and edge dislocations, respectively. *A* denotes the slip area of the edge/screw dislocation, and $\vec{b}$ is the Burgers vector of the dislocation.

**Figure S22.** Initial microstructures of commercial-purity Zr (CP-Zr) and hierarchical ultrafine-twinned Zr (UFT-Zr). a,b) Electron backscatter diffraction (EBSD) scans showing initial coarse-grain structures in CP-Zr. c-f) UFT-Zr has a hierarchical ultrafine-twinned structure. High density of deformation twins of different variants, primarily {10$\bar{\text{1}}$2} and {11$\bar{\text{2}}$1} twins, are observed in (d) and (f). The average grain size (*d_avg._*) of CP-Zr and UFT-Zr are 30.6 μm and 3.42 μm, respectively, while the average twin thickness (*t_avg._*) of UFT-Zr is only 0.84 μm.

**Figure S23.** Mechanical propertes of CP-Zr and UFT-Zr. a) Engineering tensile stress-strain curves. b) The *J*-integral-based crack resistance (*J*-R) curves. The fracture toughness *K_JIc_* for CP-Zr and UFT-Zr are 69.0 and 117.7 MPa·m^1/2^, respectively.

**Figure S24.** Cracking behaviors of CP-Zr and UFT-Zr SENB samples. a) Two-dimensional strain distribution of CP-Zr near the crack tip at crack initiation (∆*a* ≈ 0.2 mm), obtained using the DIC method on the surface (under the plane-stress conditions), shows a small plastic zone accompanied by minimal deformation. b) SEM image at the pre-polished surface reveals a small crack tip opening displacement (*CTOD*) at a crack extension of ∆*a* ≈ 2 mm. c,d) The enlarged SEM images of (b) show a non-uniform and slight deformation morphology near (c) crack profile and (d) crack tip. No obvious slip is observed within most of grains, accompanied by the formation of micro-cracks. e) Strain distribution map at crack initiation on the surface indicates a much larger plastic zone with extensive strain ahead of the crack tip for UFT-Zr. f) A higher *CTOD* is observed in UFT-Zr at the same crack extension of ∆*a* ≈ 2 mm. g,h) Severe plastic deformation occurs near the crack tip, without cracking along the twin boundaries. i,j) Optical microscope images at the mid-thickness surface (under plane-strain conditions) reveal a much higher *CTOD* for UFT-Zr compared to CP-Zr. k,l) SEM images of fracture surfaces show semi-brittle fracture mode with a mixture of cleavage facets and a few dimples in (k) CP-Zr, whereas (l) UFT-Zr exhibits a completely ductile fracture with numerous tiny dimples. The stress intensity factor (*K_i_*) at the crack tip for CP-Zr and UFT-Zr are ~ 28, 45 MPa·m^1/2^ at crack initiation (∆*a* ≈ 0.2 mm), and ~ 24, 42 MPa·m^1/2^ at a crack extension of ∆*a* ≈ 2 mm.

**Supplementary Tables**

**Table S1.** Chemical compositions (wt%) of CP-Ti, HDD-Ti, and UFT-Ti.

| Samples | Fe | C | N | O |
| --- | --- | --- | --- | --- |
| CP-Ti | 0.049 | <0.004 | <0.003 | 0.16 |
| HDD-Ti | 0.045 | 0.004 | <0.003 | 0.16 |
| UFT-Ti | 0.046 | <0.004 | 0.003 | 0.16 |

**Table S2.** Chemical compositions (wt%) of CP-Zr.

| Hf | Fe | C | N | H | O |
| --- | --- | --- | --- | --- | --- |
| 0.85 | ≤0.016 | ≤0.005 | ≤0.005 | ≤0.005 | ≤0.01 |

Table S3. Uniaxial tensile and fracture toughness properties of CP-Ti, HDD-Ti and UFT-Ti at room temperature.

| **Property** | CP-Ti | HDD-Ti | UFT-Ti |
| --- | --- | --- | --- |
| 0.2%-offset yield strength, *σ_y_* (MPa) | 389 ± 5 | 517 ± 5 | 630 ± 15 |
| Specific yield strength (MPa·cm^3^·g^-1^) | 86.4 ± 1.2 | 114.9 ± 1.1 | 140.0 ± 3.4 |
| Ultimate tensile strength, *σ_uts_* (MPa) | 490 ± 5 | 592 ± 3 | 688 ± 8 |
| Effective yield stress, *σ_0_* (MPa) | 440 ± 5 | 554 ± 2 | 659 ± 12 |
| Elongation to failure, *ε_f_* (%) | 27.4 ± 1.7 | 20.8 ± 0.9 | 18.5 ± 0.4 |
| Uniform elongation, *ε_u_* (%) | 9.9 ± 0.4 | 7.3 ± 0.3 | 6.5 ± 0.5 |
| *J*-integral at crack initiation, *J_Ic_* (kJ∙m^-2^) | 73.3 ± 2.2 | 87.9 ± 4.4 | 287.0 ± 2.0 |
| Fracture toughness at crack initiation, *K_JIc_* (MPa∙m^1/2^) | 94.2 ± 1.4 | 103.3 ± 2.6 | 186.5 ± 0.7 |
| Crack growth *J*-integral at *Δa* ~ 1.5 mm, *J_SS_* (kJ∙m^-2^) | 178.3 ± 4.9 | 246.7 ± 17.0 | 642.1 ± 11.4 |
| Crack growth toughness at *Δa* ~ 1.5 mm, *K_SS_* (MPa∙m^1/2^) | 147.0 ± 2.0 | 172.8 ± 6.0 | 278.9 ± 2.5 |
| Maximum *J*-integral, *J_max_* (kJ∙m^-2^) | 264.0 | 332.4 | 395.4 |
| 10 *J_Ic_*/*σ_0_* (mm) | 1.7 | 1.6 | 4.4 |
| *b_0_*, *B* (mm) | 6 | 6 | 6 |

Table S4. Mechanical properties of UFT-Ti compared with other metals at room temperature.

| Materials | Yield strength, (MPa) | Specific yield strength (MPa·cm^3^·g^-1^) | Crack-initiation toughness, *J_Ic_* (kJ/m^2^) | Fracture toughness, *K_JIc_* (MPa·m^1/2^) | Ref. |
| --- | --- | --- | --- | --- | --- |
| Austenitic stainless steels | 200-300 | 25-38 | 59-367 | 112-228 | 12 |
|  | 285-712 | 36-90 | 39-262 | 91-236 | 13 |
| CrCoNi | 440 | 53 | 212 | 208 | 14 |
| CrMnFeCoNi | 410 | 51 | 250 | 217 | 15 |
| V_10_Cr_10_Fe_45_Co_20_Ni_15_ | 294 | 37 | 233 | 219 | 16 |
| Ti-10V-2Fe-3Al (β-Ti) | 612-686 | 133-149 | 59-75 | 84-95 | 17 |
| Ti-13Nb-13Zr (β-Ti) | 836 | 167 | 46 | 65 | 18 |
| Ti-15Mo-5Zr-3Al (β-Ti) | 838 | 171 | 17 | 40 | 18 |
| Ti-10V-2Fe-3Al (β-Ti) | 529-718 | 115-156 | 106-133 | 102-115 | 19 |
| CP-Ti | 421-750 | 94-167 | 40-88 | 70-103 | 20 |
| CP-Ti | 297-750 | 66-167 | 9-29 | 35-58 | 21 |
| CP-Ti | 275-485 | 61-108 | 28 | 81 | 18 |
| TC4-DT | 747-916 | 170-208 | 29-95 | 60-109 | 22 |
| Ti-5Al-2.3Sn-2Zr-4Mo-4Cr | 1075-1100 | 234-239 | 49-74 | 78-96 | 23 |
| Ti-5Al-4Zr-8Mo-7V | 1198-1428 | 255-304 | 11-33 | 37-64 | 24 |
| Ti-22Al-25Nb | 873-908 | 168-175 | 6-10 | 28-36 | 25 |
| Ti–15V–3Cr–3Sn–3Al | 1100-1306 | 234-278 | 20-36 | 50-67 | 26 |
| Ti-6Al-4V | 898-932 | 204-212 | 26-30 | 57-61 | 27 |
| LO-Ti | 142 | 32 | 536 | 255 | 2 |
| Twinned Ti | 350 | 78 | / | / | 28 |
| CP-Ti (this work) | 389 | 86 | 73 | 94 | / |
| HDD-Ti (this work) | 517 | 115 | 88 | 103 | / |
| UFT-Ti (this work) | 630 | 140 | 287 | 187 | / |

Table S5. Uniaxial tensile and fracture toughness properties of CP-Zr and UFT-Zr at room temperature.

| **Property** | CP-Zr | UFT-Zr |
| --- | --- | --- |
| 0.2%-offset yield strength, *σ_y_* (MPa) | 210 ± 4 | 411 ± 2 |
| Ultimate tensile strength, *σ_uts_* (MPa) | 377 ± 8 | 476 ± 9 |
| Effective yield stress, *σ_0_* (MPa) | 294 ± 6 | 444 ± 6 |
| Elongation to failure, *ε_f_* (%) | 32.0 ± 3.0 | 16.6 ± 1.2 |
| Uniform elongation, *ε_u_* (%) | 14.4 ± 0.8 | 3.9 ± 0.7 |
| *J*-integral at crack initiation, *J_Ic_* (kJ∙m^-2^) | 45.0 ± 13.9 | 130.3 ± 18.0 |
| Fracture toughness at crack initiation, *K_JIc_* (MPa∙m^1/2^) | 69.0 ± 10.4 | 117.7 ± 8.5 |
| Crack growth *J*-integral at *Δa* ~ 1.5 mm, *J_SS_* (kJ∙m^-2^) | 90.0 ± 15.1 | 258.7 ± 30.7 |
| Crack growth toughness at *Δa* ~ 1.5 mm, *K_SS_* (MPa∙m^1/2^) | 97.7 ± 8.0 | 165.7 ± 9.9 |
| Maximum *J*-integral, *J_max_* (kJ∙m^-2^) | 176.4 | 266.4 |
| 10 *J_Ic_*/*σ_0_* (mm) | 1.5 | 2.9 |
| *b_0_*, *B* (mm) | 6 | 6 |

Table S6. Primary twinning types in HCP α-Ti and α-Zr.

| Twinning plane | Twinning direction | Shear strain | Plane normal | Angle | Tolerance |
| --- | --- | --- | --- | --- | --- |
| 10$\bar{\text{1}}$2 | $\bar{\text{1}}$011 | 0.167 | 1$\bar{\text{2}}$10 | 85° | 5° |
| 11$\bar{\text{2}}$1 | $\bar{\text{1}}\bar{\text{1}}$26 | 0.627 | 1$\bar{\text{1}}$00 | 34° | 5° |
| 11$\bar{\text{2}}$2 | $\bar{\text{1}}\bar{\text{1}}$23 | 0.225 | $\bar{\text{1}}$100 | 65° | 5° |
| 11$\bar{\text{2}}$4 | 22$\bar{\text{4}}$3 | 0.22 | 01$\bar{\text{1}}$0 | 77° | 5° |

**References**

[1] ASTM International, E1820-20 Standard Test Method for Measurement of Fracture Toughness, ASTM International, West Conshohocken, PA, USA, 2020.
[2] X. W. Zou, W. Z. Han, E. Ma, Uncovering the intrinsic high fracture toughness of titanium via lowered oxygen impurity content, *Adv. Mater.* **2024**, 36, 2408286.
[3] X. W. Zou, I. J. Beyerlein, W. Z. Han, Hierarchical nanolayered structures-enabled record-high fracture resistant zircaloy, *Acta Mater.* **2024**, 279, 120300.
[4] Y. Minonishi, S. Morozumi, H. Yoshinaga, {1122} <1123> slip in titanium, *Scr. Metall.* **1982**, 16, 427.
[5] H. Numakura, Y. Minonishi, M. Koiwa, <1123>{1011} slip in titanium polycrystals at room temperature, *Scr. Metall.* **1986**, 20, 1581.
[6] Z. X. Wu, W. A. Curtin, The origins of high hardening and low ductility in magnesium, *Nature* **2015**, 526, 62–67.
[7] Z. Wu, B. Yin, W. A. Curtin, Energetics of dislocation transformations in hcp metals, *Acta Mater.* **2016**, 119, 203–217.
[8] T. Soyez, D. Caillard, F. Onimus, E. Clouet, Mobility of <c+a> dislocations in zirconium, *Acta Mater*. **2020**, 197, 97–109.
[9] Y. Lu, Y. H. Zhang, E. Ma, W. Z. Han, Relative mobility of screw versus edge dislocations controls the ductile-to-brittle transition in metals, *Proc. Natl. Acad. Sci. USA* **2021**, 118, e2110596118.
[10] Y. H. Zhang, W. Z. Han, Dislocation source efficiency as a governing factor in the ductile-to-brittle transition of metals, *J. Mater. Sci. Technol.* **2025**, 229, 173–176.
[11] R. O. Ritchie, The conflicts between strength and toughness, *Nat. Mater.* **2011**, 10, 817–822.
[12] D. H. Cook, P. Kumar, M. I. Payne, C. H. Belcher, P. Borges, W. Wang, F. Walsh, Z. Li, A. Devaraj, M. Zhang, M. Asta, A. M. Minor, E. J. Lavernia, D. Apelian, R. O. Ritchie, Kink bands promote exceptional fracture resistance in a NbTaTiHf refractory medium-entropy alloy, *Science*, **2024**, 384, 178–184.
[13] X. R. Liu, S. D. Zhang, H. Feng, J. Wang, P. Jiang, H. B. Li, F. P. Yuan, X. L. Wu, Outstanding fracture toughness combines gigapascal yield strength in an N-doped heterostructured medium-entropy alloy, *Acta Mater.* **2023**, 255, 119079.
[14] B. Gludovatz, A. Hohenwarter, K. V. S. Thurston, H. B. Bei, Z. G. Wu, E. P. George, R. O. Ritchie, Exceptional damage-tolerance of a medium-entropy alloy CrCoNi at cryogenic temperatures, *Nat. Commun.* **2016**, 7, 10602.
[15] B. Gludovatz, A. Hohenwarter, D. Catoor, E. H. Chang, E. P. George, R. O. Ritchie, A fracture-resistant high-entropy alloy for cryogenic applications, *Science* **2014**, 345, 1153–1158.
[16] Y. H. Jo, K. Y. Doh, D. G. Kim, K. Lee, D. W. Kim, H. Sung, S. S. Sohn, D. Lee, H. S. Kim, B. Lee, S. Lee, Cryogenic-temperature fracture toughness analysis of non-equi-atomic V10Cr10Fe45Co20Ni15 high-entropy alloy, *J. Alloy. Compd.* **2019**, 809, 151864.
[17] K. Toyama, T. Maeda, The Effect of Heat Treatment on the Strength and Fracture Toughness of Ti-10V-2Fe-3Al, *Trans. Iron Steel Inst. Jpn.* **1986**, 26, 814–821.
[18] M. Niinomi, Mechanical properties of biomedical titanium alloys, *Mater. Sci. Eng. A* **1998**, 243, 231–236.
[19] A. Bhattacharjee, V. K. Varma, S. V. Kamat, A. K. Gogia, S. Bhargava, Influence of β grain size on tensile behavior and ductile fracture toughness of titanium alloy Ti-10V-2Fe-3Al, *Metall. Mater. Trans. A* **2006**, 37, 1423–1433.
[20] I. Sabirov, R. Z. Valiev, I. P. Semenova, R. Pippan, Effect of equal channel angular pressing on the fracture behavior of commercially pure titanium, *Metall. Mater. Trans. A* **2010**, 41, 727–733.
[21] Q. Li, S. Huang, Y. Zhao, Y. Gao, U. Ramamurty, Simultaneous enhancements of strength, ductility, and toughness in a TiB reinforced titanium matrix composite, *Acta Mater.* **2023**, 254, 118995.
[22] P. Guo, Y. Zhao, W. Zeng, Q. Hong, The effect of microstructure on the mechanical properties of TC4-DT titanium alloys, *Mater. Sci. Eng. A* **2013**, 563, 106–111.
[23] X. Shi, W. Zeng, Q. Zhao, The effects of lamellar features on the fracture toughness of Ti-17 titanium alloy, *Mater. Sci. Eng. A* **2015**, 636, 543–550.
[24] W. Zhu, J. Lei, B. Su, Q. Sun, The interdependence of microstructure, strength and fracture toughness in a novel β titanium alloy Ti–5Al–4Zr–8Mo–7V, *Mater. Sci. Eng. A* **782**, 139248 (2020).
[25] Y. Zheng, W. Zeng, D. Li, Q. Zhao, X. Liang, J. Zhang, X. Ma, Fracture toughness of the bimodal size lamellar O phase microstructures in Ti-22Al-25Nb (at.%) orthorhombic alloy, *J. Alloys Compd.* **2017**, 709, 511–518.
[26] P. Manikandan, K. N. Kumar, G. S. Rao, Fracture toughness of Ti–15V–3Cr–3Sn–3Al titanium alloy in different heat-treated conditions, *Trans. Indian Inst. Met.* **2019**, 72, 1507–1510.
[27] A. Gutierrez, M. Hahn, Y. J. Li, A. Dehbozorgi, W. Hohorst, M. Schwartz, J. Orlita, Y. T. Hein, N. Guanzon, X. Sun, O. S. Es-Said, The effect of different annealing conditions on the anisotropy of the fracture toughness of Ti-6Al-4V, *J. Mater. Eng. Perform.* **2019**, 28, 7155–7164.
[28] S. Zhao, R. Zhang, Q. Yu, J. Ell, R. O. Ritchie, A. M. Minor, Cryoforged nanotwinned titanium with ultrahigh strength and ductility, *Science* **2021**, 373, 1363–1368.
